# Digestive tract nematode infections in non-native invasive American mink with the first molecular identification of *Molineus patens*

**DOI:** 10.1016/j.ijppaw.2020.12.006

**Published:** 2020-12-29

**Authors:** Marta Kołodziej-Sobocińska, Małgorzata Tokarska, Hanna Zalewska, Marcin Popiołek, Andrzej Zalewski

**Affiliations:** a Mammal Research Institute, Polish Academy of Sciences, Stoczek 1, 17-230, Białowieża, Poland; b Department of Parasitology, Institute of Genetics and Microbiology, Wrocław University, Przybyszewskiego 63/77, 51-148, Wrocław, Poland

**Keywords:** Digestive tract, Genetical methods, *Neovison vison*, *Aonchotheca putorii*, *Molineus patens*

## Abstract

Parasites may negatively affect hosts condition, especially when infection intensity is high. Species introduced to a new habitat are often less exposed to a parasite pressure but may accumulate parasites in time. American mink (*Neovison vison*) introduced to Europe, Asia, and South America is an example of such invasive species. We analysed nematode prevalence and digestive tract infection intensity in 796 feral American mink from Poland. The analyses were performed separately for stomach, duodenum, small intestine and large intestine. Parasite species identification was performed using molecular methods based on highly conserved nuclear 18S rRNA gene and supplemented with morphological analysis. In total, we collected 26,852 nematodes and 98.6% of them were isolated from mink stomachs. We found positive association between infection intensity in stomach and other parts of digestive tract. Nematode prevalence was estimated at 63.8% and average infection intensity per one American mink at 52.9 (range from 1 to 1118). If the stomach results were theoretically and intentionally omitted the prevalence was 5 times lower (12.7%) and infection intensity 14 times lower (3.7; range 1–50). We identified two nematode species in digestive tracts of American mink: *Aonchotheca putorii* and *Molineus patens*. The 18S rRNA gene sequence of *Molineus patens* has been reported for the first time. The results showed that *Aonchotheca putorii* is a dominating nematode in the invasive American mink and that it inhabits stomach intensively and preferably.

## Highlights

• Parasitic infection monitoring in the wild is the important indicator of the population condition.
• *Aonchotheca putorii* is a dominating nematode in the invasive American mink.
• *Aonchotheca putorii* inhabits mink stomach intensively and preferably.
• Molecular identification of *Molineus patens* has been reported for the first time.
• The importance of using of the whole digestive tract in parasitological monitoring has been emphasized.

## Graphical abstract

**Figure:**
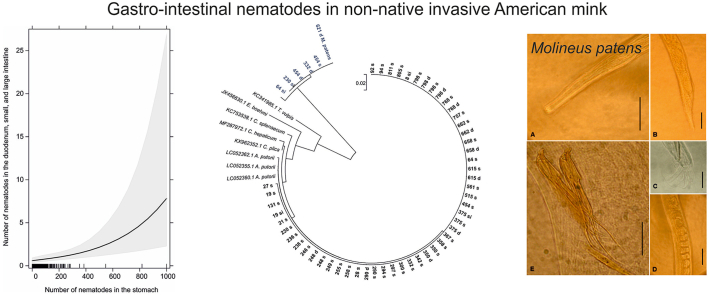

## 1 Introduction

The influence of pathogens (parasites, bacteria, or viruses) on infected hosts may vary from almost imperceptible to lethal and the effect may be related to pathogen type and infection intensity (Anderson and May 1979; Beldomenico et al., 2008; Pedersen and Fenton, 2007). Parasites may affect hosts in many ways, influencing their body condition, reproduction or mortality, altering their behaviour or vulnerability to predation, and thus affecting the status of the entire population and its susceptibility to various infections (Beldomenico et al., 2008; Gorsich et al., 2014; Kołodziej-Sobocińska, 2019; Neuhaus, 2003; Thomas et al., 1998; Turgeon et al., 2018; Winternitz et al., 2012). On the other hand, invasive non-native species (INNS) are often released from site-specific parasite pressure as they have lower number of parasites in a new range (Enemy Release Hypothesis) (Laurimaa et al., 2016; Torchin et al., 2003). Both, low parasite species richness and low parasite infection intensity in an invaded range may increase the INNS fitness (e.g. fecundity and/or survival), and accelerate their expansion. This phenomenon may generally explain the INNS success in establishing novel populations. However, the genetic variation and functional immune diversity of the INNS is shaping host-parasite relationships (Biedrzycka et al., 2020). Lower genetic variation may cause the reduction of the protective responses resulting in fast accumulation of parasites after introduction.

American mink (*Neovison vison*), one of the most invasive non-native mammals (Nentwig et al., 2010), originates from North America and has been introduced to Europe, Asia and South America both on purpose, e.g. in the former Soviet Union and accidently, through escapes from fur farms established in many countries in 1920s (Bonesi and Palazon, 2007). First feral populations in Europe were recorded in the 30s of 20th century and they have gradually colonized large parts of the continent. Colonization of new territories may potentially result in a spread of diseases carried by American mink. In many regions, where mink populations have been derived from farm escapees, they are still well supplied by ongoing introductions (Zalewski et al., 2010, 2011). American mink is an important invasive mammal species serving in its introduced range as both, intermediate and definitive host for many endoparasite species belonging to various taxonomic groups: trematodes, cestodes, nematodes (Shimalov and Shimalov, 2001a; Torres et al., 2003, 2008); among them such zoonotic parasites as: *Trichinella* spp., *Alaria* spp, *Spirometra erionaceieuropaei*, *Echinococcus* spp., *Toxocara* spp. (Hurníková et al., 2016; Kołodziej-Sobocińska et al., 2020; Kondzior et al., 2020; Shimalov and Shimalov, 2001a). Our previous study showed that the increased abundance of nematodes inhabiting digestive tract was observed with time since the American mink populations were established and, as a result, it caused a decline in mink body condition (Kołodziej-Sobocińska et al., 2018). This suggests that gastro-intestinal nematodes may have, among other parasites, important impact on mink fitness and are more pathogenic than other internal parasites such as trematodes and cestodes. However, it should be emphasized that in the cited above study all gastro-intestinal nematodes were analysed together without distinguishing particular species. Thus, to fully understand the impact of nematodes on mink, it is necessary to identify their species and to describe their distribution along digestive tracts.

The aim of our study was to identify and estimate infection parameters of nematode species in digestive tracts of American mink individuals, using morphological and molecular methods and to report the nematodes distribution in the subsequent parts of studied digestive tracts. Reliable and comparable methods of parasite identification and counting are crucial for estimating the effect of pathogens on wild mammalian hosts, especially those non-native invasive ones. That would enable the proper management and controlling their influence on native fauna.

## 2 Material and methods

### 2.1 American mink carcasses and parasite collection

We collected 796 American mink in 2005–2017 from Poland. American mink were trapped and then removed from collection sites located within the scope of bird conservation projects, under permission granted by local and government authorities. American mink carcasses were frozen and stored at −20 °C before dissection. Then, their digestive tracts were extracted. Each digestive tract was divided into 4 parts: stomach (ST), duodenum (D), small intestine (SI), large intestine (LI) and all isolated parasites were counted for every part individually. In each part of the digestive tract, we separately studied 3 groups of parasites: 1) nematodes; 2) trematodes; and 3) cestodes. We analysed the associations between the number of nematodes in the stomach and other parts of the digestive tract (duodenum, small intestine, and large intestine) using the generalised linear model (GLM) with quasipoisson distribution.

### 2.2 Genetic and morphological identification of parasites

#### 2.2.1 DNA extraction

Nematode individuals were extracted from the stomach, duodenum, small intestine and large intestine, using the digestive tract content decanting method. After initial visual identification nematodes were preserved in 70% ethyl ethanol and 80 specimens were randomly selected for detailed genetic analyses. Each sample consisting of 1–6 parasite individuals (collected from one host and a particular part of the digestive tract; see Appendix 1, Table S1) was put into a 1.5 ml Eppendorf tube with 300 μl of sterile water. The samples were frozen at −80 °C for several weeks. Then, part of the samples were thawed, sonicated (Sonic Ruptor 400 OMINI INTERNATIONAL) and then frozen again at −80 °C. The other parts of the samples were thawed to 98 °C and then frozen to −80 °C in three subsequent cycles, each lasting approx. 24 h. After the last thaw, the samples remained frozen at −80 °C until needed for further procedures. The DNA was extracted in accordance with the Sherlock AX (A&A Biotechnology) protocol, only that the DNA precipitate was suspended in 20 μl of TE buffer (supplied).

#### 2.2.2 Morphological identification

Five specimens isolated from the duodenum (1 male and 4 females) – visually different from the rest – were cleared in lactophenol, measured and photographed (see Appendix 1, Fig. S1). The morphological features were described in accordance with the publications of Durette-Desset and Pesson (1987), Kozlov (1977), Marroquin-Mucino et al. (2017), Popiołek et al. (2009). After the morphological identification, from three of these specimens DNA was extracted in accordance with the described above method and used for genetic analyses.

#### 2.2.3 18S rRNA analysis

The nuclear 18S rRNA gene is highly conserved among the different species and thus serves well for phylogenetic assays and comparisons (Yan et al., 2013). We used the pair of primers 18S 965 and 18S 1573R (Powers et al., 2009) for the molecular identification of the samples obtained as well as for the comparison of the samples acquired from different parts of the digestive tract. PCR was performed using HotStarTaq Master Mix (QIAGEN), following the manual, except that the reaction volume was decreased to 10 μl, with 20 pmol of each primer and 20–40 ɳg of DNA. The PCR conditions were the same as described in (Guardone et al., 2013). The sequences were run and read on genetic Analyzer 3130xl (Life Technologies). We applied the multiple alignment tool in the BioEdit Sequence Alignment Editor (Hall, 1999) for comparisons of the sequences and to juxtapose them with other evolutionarily close species: *Aonchotheca putorii* (avians, Japan, LC052355, LC052360.1, LC052362), *Eucoleus boehmi* (JX456630.1), *Capillaria plica* (KX962352.1), *Calodium hepaticum* (MF287972.1), *Calodium splenaecum* (KC753538.1), *Trichuris vulpis* (KC341985.1). MEGA6 software was used to build the phylogenetic tree (Tamura et al., 2013).

## 3 Results

### 3.1 Nematodes in American mink

**Fig. 1:**
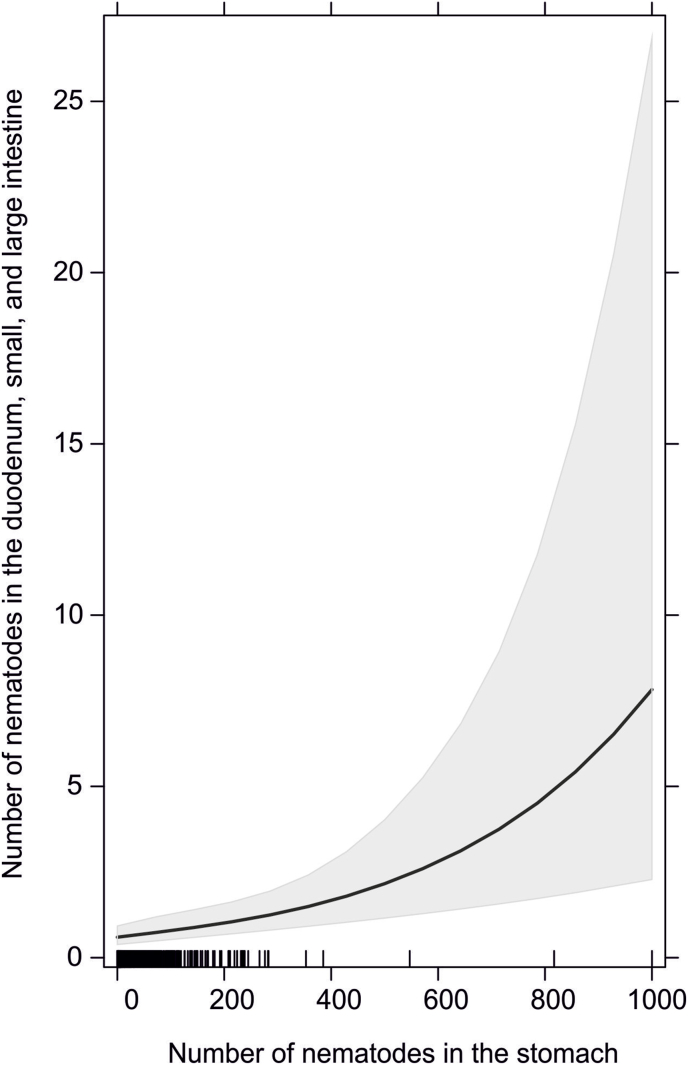
Relation between the number of nematodes in the stomach of American mink and the number of nematodes in other parts of the digestive tract (duodenum, small intestine, and large intestine).

In total, we collected 26,852 nematodes from 796 feral American mink. The majority – 98.6% (26,476) had been isolated from stomachs, 0.7% (185) duodenum, 0.6% (170) from small intestine and only 0.1% (21) from large intestine (Table 1). The total number of nematodes found in the duodenum, small intestine, and large intestine was associated to the number of parasites present in stomach (GLM, Estimate = 0.0025, SE = 0.00069, p = 0.0002). The number of nematodes in duodenum, small intestine, and large intestine increased from 0.5 (CI = 0.38–0.93) with no nematodes in the stomach, to up to 7.8 (CI = 2.28–26.84) with 1000 nematodes found in the stomach (Fig. 1).

**Table 1:** Comparison of nematode infection parameters and nematode distribution along digestive tract taking into account the parts of the analysed digestive tract of feral American mink (N = 796). ST – stomach; D – duodenum; SI – small intestine; LI – large intestine; prevalence – proportion of infected American mink among the total number of analysed animals; mean infection intensity – the total number of nematodes divided by the number of infected American mink; nematode proportion – percentage of nematodes found in particular part of digestive tract.

Digestive tract parts	Infection parameters		Nematode distribution	
	Prevalence (%)	Mean infection intensity (range)	Number of nematodes	Nematode proportion
all (ST + D + SI + LI)	63.8	52.9 (1–1118)	26,852	100
ST	62.4	53.3 (1–1118)	26,476	98.6
D	9.0	2.6 (1–26)	185	0.7
SI	4.5	4.8 (1–50)	170	0.6
LI	1.0	1.1 (1–13)	21	0.1
D + SI + LI	12.7	3.7 (1–50)	376	1.4

With the whole digestive tract analysed, the nematode prevalence was five times higher than when the stomach was excluded from the study (63.8 and 12,7% respectively) (Table 1). Differences in estimated infection intensity were much more pronounced: 52.9 (range 1–1118) nematodes per one American mink on average when the complete digestive tract was analysed and only 3.7 (range 1–50) nematodes per one mink when the results from the stomachs were excluded, which is over 14 times less (Table 1). This comparison revealed that nematodes in American mink inhabit mainly stomach.

### 3.2 Parasite species identification

**Fig. 2:**
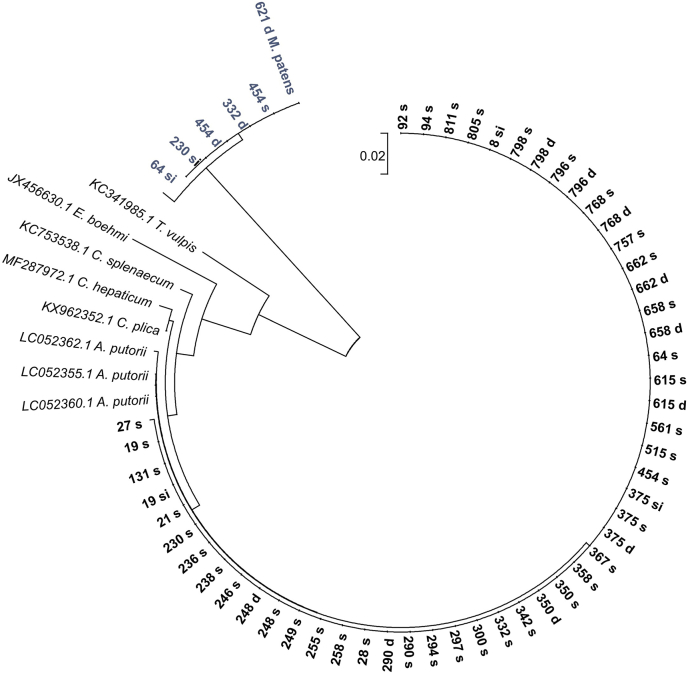
A phylogram based on the small subunit of 18S rRNA nuclear gene fragments for the 58 sequences obtained from the parasites isolated from different parts of the American mink digestive tract: s – stomach; d – duodenum; si – small intestine (bold – *Aonchtheca putorii*, navy blue and bold – *Molineus patens*) and sequences achieved from GeneBank (http://www.ncbi.nlm.nih.gov/) (in italics) of the other 8 Trichuridae species (phylum Nematoda). All newly achieved sequences are in bold (details in Appendix 1, Fig. S2). The evolutionary relationships of the taxa were implied using the maximum likelihood estimation method (Tamura and Nei, 1993) embedded in MEGA6 software (Tamura et al., 2013). The tree with the highest log likelihood (−1262.9129) is shown. The evolutionary distances were computed using the maximum composite likelihood method (Tamura et al., 2004) and are expressed as a number of base substitutions per site. All positions containing gaps and missing data were eliminated. There were a total of 456 positions in the final dataset.

Molecular and morphological analyses of the nematodes revealed that they belonged to two species: *Aonchotheca putorii* and *Molineus patens* (Fig. 2; Appendix 1, Table 1, Fig. S1, Fig. S2). Based on genetic analyses, *A. putorii* was found in all samples from the stomach and also in 10 samples from the duodenum and 3 samples from the intestine (Fig. 2; Appendix 1, Table S1, Fig. S2). They showed no genetic divergence either within the same host or between hosts, even if their localizations were relatively distant geographically (Fig. 2; Appendix 1, Fig. S2). Comparing the sequences obtained from American mink to available sequences from GenBank, the maximum likelihood analyses (Tamura and Nei, 1993) grouped all the samples obtained from American mink and one sample of *Aonchotheca putorii* from GenBank (LC052360.1) in one monophyletic group (Fig. 2). Based on the available data reference in GenBank (using the BLAST tool), we could not identify the 18S rRNA gene sequences of six samples: 1 sample collected from the stomach, 3 samples from the duodenum, and 2 samples from the small intestine (Appendix 1, Table S1); all these sequences were grouped in one monophyletic group, but they were distinct from *Aonchotheca putorii* or other compared species (Fig. 2). Based on morphological examination and measurements, the samples were identified as *Molineus patens* Dujardin, 1845 (Durette-Desset and Pesson, 1987; Kozlov, 1977; Marroquin-Mucino et al., 2017; Popiołek et al., 2009). The main diagnostic features are: the shape and dimensions of the spicules and gubernaculum, the pattern and number of synlophe ridges and the arrangement of the ribs in the bursa copulatrix in the male (see Appendix 1, Fig. S1). The obtained sequences of 18S rRNA have been deposited in GeneBank with accession numbers: MT431688 and MW397199-MW397204 for *Molineus patens* and MW386903 to MW386953 for *Aonchotheca putorii* (syn. *Capillaria putorii*) (see details in Appendix 1, Table S1).

## 4 Discussion

Our study showed that the American mink in Poland is predominantly infected with two gastro-intestinal nematode species – *Aonchotheca putorii* and *Molineus patens*. Almost all nematodes were found in stomach and the dominating species was *A. putorii*. For the first time we obtained 18S rRNA gene sequences of *M. patens* appropriate for the molecular identification of this species.

In total, we collected a huge number of almost 27,000 nematode specimens. As molecular analysis of all collected nematodes was not feasible, we chose randomly 80 nematodes from each part of the digestive tract for molecular identification of the parasite species content. Our data revealed that almost 99% of nematodes were found in stomachs and that except one specimen examined genetically – all the rest extracted from stomachs belonged to *A. putorii* species. We presume that a large number of nematodes in stomach (even over 1000 specimens in one mink host) may possibly damage the gastric epithelial tissue, cause digestive disorders and, consequently, limit the absorption of nutrients. Only scarce data on pathogenicity of *A. putorii* are available and they concern infections of domestic cats where chronic gastritis, vomiting, diarrhoea, and weight loss were observed (Curtsinger et al., 1993; Nakanishi and Nakanishi, 2013). It is acknowledged that other nematode species inhabiting stomach in wildlife animals are also responsible for numerous lesions in infected hosts. Granulomatous gastric nodules, characterized by marked fibrosis and inflammatory infiltrate of lymphocytes, plasma cells, eosinophils, and macrophages surrounding *Spirocerca vulpis* nematodes parasitizing the stomach wall were observed in red fox in Portugal (Gama et al., 2020) while stomach scars or extensive fibrosis putatively associated with *Anisakis* spp. nematodes caused ulcerative lesions that may compromise the health of the cetaceans in the north-east Atlantic (Pons-Bordas et al., 2020).

The second species found in American mink in this study is *Molineus patens.* Selected genotyped samples showed only one specimen of *M. patens* in the stomach while the rest were found in the intestine. This is concordant with other studies showing that *A. putorii* much more often inhabits stomach whereas, *M. patens* inhabits mainly the intestine (Kretschmar, 2016; Martínez-Rondán et al., 2017; Nugaraite et al., 2014, 2019; Torres et al., 2004). Both species are commonly detected nematodes in Mustelid hosts and were found in seven species: American mink, European mink (*Mustela lutreola*), pine marten (*Martes martes*), stone marten (*Martes foina*), European polecat (*Mustela putorius*), sable (*Martes zibellina*), American marten (*Martes americana*) (Anisimova, 2004; Foreyt and Lagerquist, 1993; Nugaraite et al., 2019; Odnokurtsev and Sedalischev, 2011; Torres et al., 2004, 2008). *M. patens* was detected not only in Mustelidae but also in Canidae: red fox *Vulpes vulpes,* raccoon dog *Nyctereutes procyonoides,* dog *Canis familiaris*, Gliridae: *Eliomys quercinus*, and Ursidae – *Ursus arctos* (Borgsteede, 1984; Durette-Desset and Pesson, 1987; Jones et al., 1980; Kozlov, 1963, 1977; Rataj et al., 2013; Rausch et al., 1979; Shimalov and Shimalov, 2001b; Thiess et al., 2001).

As nematodes in mink inhabit mainly stomach, accidental and/or unintentional excluding stomach from the analyses may lead to significant decrease of all infection indexes of nematodes inhabiting digestive tracts of hosts. Stomach may be excluded from parasitological analyses due to e.g. simultaneous studies on host diet composition (Anisimova 2002). As mentioned above, the prevalence of nematodes was 5 times lower and infection intensity even 14 times lower than actual when the stomach was deliberately excluded from the analyses. We found a positive correlation between nematode infection intensity in the stomach and other parts of the digestive tract in the American mink, which suggests that with an increase of infection intensity in the stomach, nematode specimens may also be found in other parts of the digestive tract, e.g. the duodenum or small intestine.

Many studies rely on the prevalence and the number of parasites collected from the digestive tracts of definitive hosts and use these parameters to assess the influence of the parasites on host condition, fecundity, mortality and, in general, on host population size (Gorsich et al., 2014; Neuhaus, 2003; Stien et al., 2002; Winternitz et al., 2012). Part of these studies showed that parasites may have a deleterious effect on the host (Anderson and May 1979; Beldomenico et al., 2008). However, parasites differ in their pathogenicity, e.g. some may negatively affect fitness and body condition, but others may not (Kołodziej-Sobocińska et al., 2018). Thus, irrefutable molecular identification of the parasite species and reliable estimation of the number of parasites infecting specific host are very important to predict the effects of parasite influence on both, the host and the population. Unfortunately, the genetic and genomic data on many parasitic species is still scarce. It is of great importance to apply both, molecular and morphological tools for reliable identification of parasitic species.

## 5 Conclusion

To conclude, we molecularly identified two nematode species: *A. putorii* and *M. patens*, both inhabiting specific parts of digestive tract of an invasive American mink. Gastro-intestinal nematodes in the American mink and other mustelids inhabit mostly stomachs, therefore we emphasize the importance of using of the whole digestive tract (including stomach) in parasitological monitoring of mammalian hosts. Disease monitoring in the wild is one of the important indicators of the population condition. Thus, to establish actions for the proper management of native, non-native invasive, as well as endangered species we should be able to obtain the appropriate indicators of the health status of monitored species in time and/or space.

## Author contributions

**Marta Kołodziej-Sobocińska**: Conceptualization, Methodology, Investigation, Resources, Writing - Original Draft, Writing - Review & Editing, Visualization, Supervision **Małgorzata Tokarska**: Methodology, Formal analysis, Writing - Review & Editing, Visualization **Hanna Zalewska:** Investigation **Marcin Popiołek:** Methodology, Investigation, Writing - Review & Editing, Visualization **Andrzej Zalewski:** Conceptualization, Methodology, Formal analysis, Resources, Data Curation, Writing - Original Draft, Writing - Review & Editing, Visualization, Funding acquisition.

## Declaration of competing interest

The authors declare that they have no known competing financial interests or personal relationships that could have appeared to influence the work reported in this paper.
