# Supplementary material for: Digestive tract nematode infections in non-native invasive American mink with the first molecular identification of Molineus patens
Source: Int J Parasitol Parasites Wildl. 2020 Dec 29;14:48–52. doi: 10.1016/j.ijppaw.2020.12.006 (PMC7787951; doi:10.1016/j.ijppaw.2020.12.006)
Supplement: Multimedia component 1 [file mmc1.docx]

**Appendix 1**

**Title:** Digestive tract nematode infections in non-native invasive American mink (*Neovison vison*) with the first molecular identification of *Molineus patens*

**Authors:** Marta Kołodziej-Sobocińska^1^, Małgorzata Tokarska^1^, Hanna Zalewska^1^, Marcin Popiołek^2^, Andrzej Zalewski^1*^

^1^Mammal Research Institute, Polish Academy of Sciences, Stoczek 1, 17-230 Białowieża, Poland; ^2^Department of Parasitology, Institute of Genetics and Microbiology, Wrocław University, Przybyszewskiego 63/77, 51-148 Wrocław, Poland

Table S1. Information on the analyzed samples from 6 localities and various parts of digestive tract. Shaded boxes mean no samples collected from particular part of digestive tract. GeneBank accession numbers of all sequences are shown in brackets. Three samples classified morphologically are also denoted with the letter ‘m’. WMNP stands for – Warta Mouth National Park, DNP – Drawa National Park, SNP – Słowiński National Park, NNP – Narew National Park, BNP – Biebrza National Park and VR for Vistula River.

|  | Sample ID-localization | Stomach (s) | Duodenum (d) | Small intestine (si) |
| --- | --- | --- | --- | --- |
|  | 8-DNP |  |  | *A. putorii* (MW386949) |
|  | 19-WMNP | *A. putorii* (MW386904) |  | *A. putorii* (MW386905) |
|  | 21-DNP | *A. putorii* (MW386906) |  |  |
|  | 27-DNP | *A. putorii* (MW386916) |  |  |
|  | 28-DNP | *A. putorii* (MW386917) |  |  |
|  | 64- WMNP | *A. putorii* (MW386937) |  | *M. patens* (MW397204) |
|  | 92-SNP | *A. putorii* (MW386952) |  |  |
|  | 94-SNP | *A. putorii* (MW386953) |  |  |
|  | 131-BNP | *A. putorii* (MW386903) |  |  |
|  | 230-BNP | *A. putorii* (MW386906) |  | *M. patens* (MW397199) |
|  | 236-SNP | *A. putorii* (MW386908) |  |  |
|  | 238-BNP | *A. putorii* (MW386909) |  |  |
|  | 246-VR | *A. putorii* (MW386910) |  |  |
|  | 248-VR | *A. putorii* (MW386912) | *A. putorii* (MW386911) |  |
|  | 249-VR | *A. putorii* (MW386913) |  |  |
|  | 255-VR | *A. putorii* (MW386914) |  |  |
|  | 258-VR | *A. putorii* (MW386915) |  |  |
|  | 290-NNP | *A. putorii* (MW386919) | *A. putorii* (MW386918) |  |
|  | 294-WMNP | *A. putorii* (MW386920) |  |  |
|  | 297-BNP | *A. putorii* (MW386921) |  |  |
|  | 300-WMNP | *A. putorii* (MW386922) |  |  |
|  | 332-BNP | *A. putorii* (MW386923) | *M. patens* (MT431688, m) |  |
|  | 342-SNP | *A. putorii* (MW386924) |  |  |
|  | 350-SNP | *A. putorii* (MW386926) | *A. putorii* (MW386925) |  |
|  | 358-NNP | *A. putorii* (MW386927) |  |  |
|  | 367-DNP | *A. putorii* (MW386928) |  |  |
|  | 375-NNP | *A. putorii* (MW386930) | *A. putorii* (MW386929) | *A. putorii* (MW386931) |
|  | 454-NNP | *A. putorii(*MW386932*), M. patens* (MW397202) | *M. patens* (MW397200, m) |  |
|  | 515-SNP | *A. putorii* (MW386933) |  |  |
|  | 561-DNP | *A. putorii* (MW386934) |  |  |
|  | 615-BNP | *A. putorii* (MW386936) | *A. putorii* (MW386935) |  |
|  | 621-BNP |  | *M. patens* (MW397203, m) |  |
|  | 658-NNP | *A. putorii* (MW386939) | *A. putorii* (MW386938) |  |
|  | 662-NNP | *A. putorii* (MW386941) | *A. putorii* (MW386940) |  |
|  | 757-NNP | *A. putorii* (MW386942) |  |  |
|  | 768-NNP | *A. putorii* (MW386944) | *A. putorii* (MW386943) |  |
|  | 796-NNP | *A. putorii* (MW386946) | *A. putorii* (MW386945) |  |
|  | 798-WMNP | *A. putorii* (MW386948) | *A. putorii* (MW386947) |  |
|  | 805-WMNP | *A. putorii* (MW386950) |  |  |
|  | 811-WMNP | *A. putorii* (MW386951) |  |  |

Figure S1. *Molineus patens* (Dujardin, 1845), A – anterior end of male (lateral view, scale bar: 100 μm), B – posterior end of female (lateral view, scale bar: 100 μm), C – posterior end of male: caudal bursa with rays (dorso-lateral view, scale bar: 100 μm); D – females with a fragment of ovijector with eggs (scale bar: 100 μm); E – male: characteristic shaped spicules with 3 terminal processes of different lengths (two short and one long) and a fragment of gubernaculum (scale bar: 50 μm).


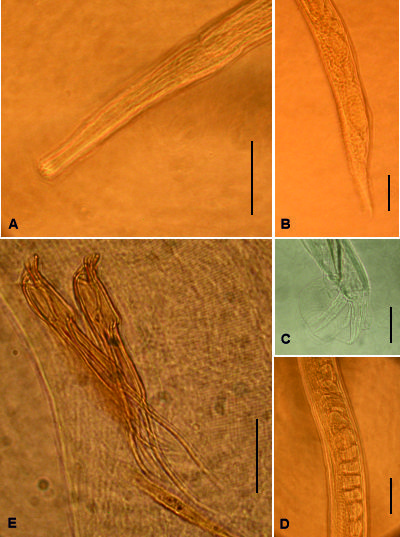


Figure S2. 18S ribosomal RNA gene sequence alignment for *Aonchotheca putorii* (from 131s to 94s) and *Molineus patens* (from 230si to 621d male) DNA samples extracted from American mink, juxtaposed with 6 GeneBank achieved sequences of 6 of other Trichuroidea (phylum Nematoda) species, achieved from GeneBank: LC052360.1 *Aonchotheca putorii*, JX456630.1 *Eucoleus boehmi*, KX962352.1 *Capillaria plica*, MF287972.1 *Calodium hepaticum*, KC753538.1 *Calodium splenaecum*, KC341985.1 *Trichuris vulpis*. s - stomach, d - duodenum, si - small intestine.

10 20 30 40 50 60 70 80 90

....|....| ....|....| ....|....| ....|....| ....|....| ....|....| ....|....| ....|....| ....|....|

131 s GTCTTTCGGT TCCGGGGGAA -GTATGG-TT GCAAAGCTGA AA-CTTAAAG GAATTGACGG AAGGG-CAC- CAC----CAG GAGTGG-AGC

19 s GTCTTTCGGT TCCGGGGGAA -GTATGG-TT GCAAAGCTGA AA-CTTAAAG GAATTGACGG AAGGG-CAC- CAC----CAG GAGTGG-AGC

19 si GTCTTTCGGT TCCGGGGGAA -GTATGG-TT GCAAAGCTGA AA-CTTAAAG GAATTGACGG AAGGG-CAC- CAC----CAG GAGTGG-AGC

21 s GTCTTTCGGT TCCGGGGGAA -GTATGG-TT GCAAAGCTGA AA-CTTAAAG GAATTGACGG AAGGG-CAC- CAC----CAG GAGTGG-AGC

230 s GTCTTTCGGT TCCGGGGGAA -GTATGG-TT GCAAAGCTGA AA-CTTAAAG GAATTGACGG AAGGG-CAC- CAC----CAG GAGTGG-AGC

236 s GTCTTTCGGT TCCGGGGGAA -GTATGG-TT GCAAAGCTGA AA-CTTAAAG GAATTGACGG AAGGG-CAC- CAC----CAG GAGTGG-AGC

238 s GTCTTTCGGT TCCGGGGGAA -GTATGG-TT GCAAAGCTGA AA-CTTAAAG GAATTGACGG AAGGG-CAC- CAC----CAG GAGTGG-AGC

246 s GTCTTTCGGT TCCGGGGGAA -GTATGG-TT GCAAAGCTGA AA-CTTAAAG GAATTGACGG AAGGG-CAC- CAC----CAG GAGTGG-AGC

248 d GTCTTTCGGT TCCGGGGGAA -GTATGG-TT GCAAAGCTGA AA-CTTAAAG GAATTGACGG AAGGG-CAC- CAC----CAG GAGTGG-AGC

248 s GTCTTTCGGT TCCGGGGGAA -GTATGG-TT GCAAAGCTGA AA-CTTAAAG GAATTGACGG AAGGG-CAC- CAC----CAG GAGTGG-AGC

249 s GTCTTTCGGT TCCGGGGGAA -GTATGG-TT GCAAAGCTGA AA-CTTAAAG GAATTGACGG AAGGG-CAC- CAC----CAG GAGTGG-AGC

255 s GTCTTTCGGT TCCGGGGGAA -GTATGG-TT GCAAAGCTGA AA-CTTAAAG GAATTGACGG AAGGG-CAC- CAC----CAG GAGTGG-AGC

258 s GTCTTTCGGT TCCGGGGGAA -GTATGG-TT GCAAAGCTGA AA-CTTAAAG GAATTGACGG AAGGG-CAC- CAC----CAG GAGTGG-AGC

27 s GTCTTTCGGT TCCGGGGGAA -GTATGG-TT GCAAAGCTGA AA-CTTAAAG GAATTGACGG AAGGG-CAC- CAC----CAG GAGTGG-AGC

28 s GTCTTTCGGT TCCGGGGGAA -GTATGG-TT GCAAAGCTGA AA-CTTAAAG GAATTGACGG AAGGG-CAC- CAC----CAG GAGTGG-AGC

290 d GTCTTTCGGT TCCGGGGGAA -GTATGG-TT GCAAAGCTGA AA-CTTAAAG GAATTGACGG AAGGG-CAC- CAC----CAG GAGTGG-AGC

290 s GTCTTTCGGT TCCGGGGGAA -GTATGG-TT GCAAAGCTGA AA-CTTAAAG GAATTGACGG AAGGG-CAC- CAC----CAG GAGTGG-AGC

294 s GTCTTTCGGT TCCGGGGGAA -GTATGG-TT GCAAAGCTGA AA-CTTAAAG GAATTGACGG AAGGG-CAC- CAC----CAG GAGTGG-AGC

297 s GTCTTTCGGT TCCGGGGGAA -GTATGG-TT GCAAAGCTGA AA-CTTAAAG GAATTGACGG AAGGG-CAC- CAC----CAG GAGTGG-AGC

300 s GTCTTTCGGT TCCGGGGGAA -GTATGG-TT GCAAAGCTGA AA-CTTAAAG GAATTGACGG AAGGG-CAC- CAC----CAG GAGTGG-AGC

332 s GCCCTTTGTA ACCGGGGGAA -GTATGG-TT GCAAAGCTGA AA-CTTAAAG GAATTGACGG AAGGG-CAC- CAC----CAG GAGTGG-AGC

342 s GTCTTTCGGT TCCGGGGGAA -GTATGG-TT GCAAAGCTGA AA-CTTAAAG GAATTGACGG AAGGG-CAC- CAC----CAG GAGTGG-AGC

350 d GTCTTTCGGT TCCGGGGGAA -GTATGG-TT GCAAAGCTGA AA-CTTAAAG GAATTGACGG AAGGG-CAC- CAC----CAG GAGTGG-AGC

350 s GTCTTTCGGT TCCGGGGGAA -GTATGG-TT GCAAAGCTGA AA-CTTAAAG GAATTGACGG AAGGG-CAC- CAC----CAG GAGTGG-AGC

358 s GTCTTTCGGT TCCGGGGGAA -GTATGG-TT GCAAAGCTGA AA-CTTAAAG GAATTGACGG AAGGG-CAC- CAC----CAG GAGTGG-AGC

367 s GTCTTTCGGT TCCGGGGGAA -GTATGG-TT GCAAAGCTGA AA-CTTAAAG GAATTGACGG AAGGG-CAC- CAC----CAG GAGTGG-AGC

375 d GTCTTTCGGT TCCGGGGGAA -GTATGG-TT GCAAAGCTGA AA-CTTAAAG GAATTGACGG AAGGG-CAC- CAC----CAG GAGTGG-AGC

375 s GTCTTTCGGT TCCGGGGGAA -GTATGG-TT GCAAAGCTGA AA-CTTAAAG GAATTGACGG AAGGG-CAC- CAC----CAG GAGTGG-AGC

375 si GTCTTTCGGT TCCGGGGGAA -GTATGG-TT GCAAAGCTGA AA-CTTAAAG GAATTGACGG AAGGG-CAC- CAC----CAG GAGTGG-AGC

454 s GTCTTTCGGT TCCGGGGGAA -GTATGG-TT GCAAAGCTGA AA-CTTAAAG GAATTGACGG AAGGG-CAC- CAC----CAG GAGTGG-AGC

516 s GTCTTTCGGT TCCGGGGGAA -GTATGG-TT GCAAAGCTGA AA-CTTAAAG GAATTGACGG AAGGG-CAC- CAC----CAG GAGTGG-AGC

561 s GTCTTTCGGT TCCGGGGGAA -GTATGG-TT GCAAAGCTGA AA-CTTAAAG GAATTGACGG AAGGG-CAC- CAC----CAG GAGTGG-AGC

615 d GTCTTTCGGT TCCGGGGGAA -GTATGG-TT GCAAAGCTGA AA-CTTAAAG GAATTGACGG AAGGG-CAC- CAC----CAG GAGTGG-AGC

615 s GTCTTTCGGT TCCGGGGGAA -GTATGG-TT GCAAAGCTGA AA-CTTAAAG GAATTGACGG AAGGG-CAC- CAC----CAG GAGTGG-AGC

64 s GTCTTTCGGT TCCGGGGGAA -GTATGG-TT GCAAAGCTGA AA-CTTAAAG GAATTGACGG AAGGG-CAC- CAC----CAG GAGTGG-AGC

658 d GTCTTTCGGT TCCGGGGGAA -GTATGG-TT GCAAAGCTGA AA-CTTAAAG GAATTGACGG AAGGG-CAC- CAC----CAG GAGTGG-AGC

658 s GTCTTTCGGT TCCGGGGGAA -GTATGG-TT GCAAAGCTGA AA-CTTAAAG GAATTGACGG AAGGG-CAC- CAC----CAG GAGTGG-AGC

662 d GTCTTTCGGT TCCGGGGGAA -GTATGG-TT GCAAAGCTGA AA-CTTAAAG GAATTGACGG AAGGG-CAC- CAC----CAG GAGTGG-AGC

662 s GTCTTTCGGT TCCGGGGGAA -GTATGG-TT GCAAAGCTGA AA-CTTAAAG GAATTGACGG AAGGG-CAC- CAC----CAG GAGTGG-AGC

757 s GTCTTTCGGT TCCGGGGGAA -GTATGG-TT GCAAAGCTGA AA-CTTAAAG GAATTGACGG AAGGG-CAC- CAC----CAG GAGTGG-AGC

768 d GTCTTTCGGT TCCGGGGGAA -GTATGG-TT GCAAAGCTGA AA-CTTAAAG GAATTGACGG AAGGG-CAC- CAC----CAG GAGTGG-AGC

768 s GTCTTTCGGT TCCGGGGGAA AGTATGG-TT GCAAAGCTGA AAACTTAAAG GAATTGACGG AAGGG-CAC- CAC----CAG GAGTGG-AGC

796 d GTCTTTCGGT TCCGGGGGAA -GTATGG-TT GCAAAGCTGA AA-CTTAAAG GAATTGACGG AAGGG-CAC- CAC----CAG GAGTGG-AGC

796 s GTCTTTCGGT TCCGGGGGAA -GTATGG-TT GCAAAGCTGA AA-CTTAAAG GAATTGACGG AAGGG-CAC- CAC----CAG GAGTGG-AGC

798 d GTCTTTCGGT TCCGGGGGAA -GTATGG-TT GCAAAGCTGA AA-CTTAAAG GAATTGACGG AAGGG-CAC- CAC----CAG GAGTGG-AGC

798 s GTCTTTCGGT TCCGGGGGAA -GTATGG-TT GCAAAGCTGA AA-CTTAAAG GAATTGACGG AAGGG-CAC- CAC----CAG GAGTGG-AGC

8 si GTCTTTCGGT TCCGGGGGAA -GTATGG-TT GCAAAGCTGA AA-CTTAAAG GAATTGACGG AAGGG-CAC- CAC----CAG GAGTGG-AGC

805 s GTCTTTCGGT TCCGGGGGAA -GTATGG-TT GCAAAGCTGA AA-CTTAAAG GAATTGACGG AAGGG-CAC- CAC----CAG GAGTGG-AGC

811 s GTCTTTCGGT TCCGGGGGAA -GTATGG-TT GCAAAGCTGA AA-CTTAAAG GAATTGACGG AAGGG-CAC- CAC----CAG GAGTGG-AGC

92 s GTCTTTCGGT TCCGGGGGAA -GTATGG-TT GCAAAGCTGA AA-CTTAAAG GAATTGACGG AAGGG-CAC- CAC----CAG GAGTGG-AGC

94 s GTCTTTCGGT TCCGGGGGAA -GTATGG-TT GCAAAGCTGA AA-CTTAAAG GAATTGACGG AAGGG-CAC- CAC----CAG GAGTGG-AGC

230 si GTCTTTCGGT TCCTGGGG-T AGTATGG-TT GCAAAGCTGA AA-CTTAAAG AAATTGACGG AATGG-CAC- CAC----CAG GAGTGG-AGC

454 d GTCTTTCGGT TCCTGGGG-T AGTATGG-TT GCAAAGCTGA AA-CTTAAAG AAATTGACGG AATGG-CAC- CAC----CAG GAGTGG-AGC

332 d GTCTTTCGGT TCCTGGGG-T AGTATGG-TT GCAAAGCTGA AA-CTTAAAG AAATTGACGG AATGG-CAC- CAC----CAG GAGTGG-AGC

454 s GTCTTTCGGT TCCTGGGG-T AGTATGG-TT GCAAAGCTGA AA-CTTAAAG AAATTGACGG AATGG-CAC- CAC----CAG GAGTGG-AGC

621 d GTCTTTCGGT TCCTGGGG-T AGTATGG-TT GCAAAGCTGA AA-CTTAAAG AAATTGACGG AATGG-CAC- CAC----CAG GAGTGG-AGC

64 si GTCTTTCGGT TCCTGGGG-T AGTATGG-TT GCAAAGCTGA AA-CTTAAAG AAATTGACGG AATGG-CAC- CAC----CAG GAGTGG-AGC

621 d male M. patens GTCTTTCGGT TCCTGGGG-T AGTATGG-TT GCAAAGCTGA AA-CTTAAAG AAATTGACGG AATGG-CAC- CAC----CAG GAGTGG-AGC

LC052360.1 A. putorii ---------- ---------- -GTATGG-TT GCAAAGCTGA AA-CTTAAAG GAATTGACGG AAGGG-CAC- CAC----CAG GAGTGG-AGC

JX456630.1 E. boehmi ---------- ---------- -GTACGG-TT GCAAAGCTGA AA-CTTAAAG GAATTGACGG AAGGG-CAC- CAC----CAG GAGTGG-AGC

KX962352.1 C. plica ---------- ---------- -GTATGG-TT GCAAAGCTGA AA-CTTAAAG GAATTGACGG AAGGG-CAC- CAC----CAG GAGTGG-AGC

MF287972.1 C. hepaticum ---------- ---------- -GTATGG-TT GCAAAGCTGA AA-CTTAAAG GAATTGACGG AAGGG-CAC- CAC----CAG GAGTGG-AGC

KC753538.1 C. splenaecum ---------- ---------- -GTATGG-TT GCAAAGCTGA AA-CTTAAAG GAATTGACGG AAGGG-CAC- CAC----CAG GAGTGG-AGC

KC341985.1 T. vulpis ---------- ---------- -GTATGG-TT GCAAAGCTGA AA-CTTAAAG GAATTGACGG AAGGG-CAC- CAC----CAG GAGTGG-AGC

100 110 120 130 140 150 160 170 180

....|....| ....|....| ....|....| ....|....| ....|....| ....|....| ....|....| ....|....| ....|....|

131 s ATGCGGCTT- AATTTGACTC AACAC--GGG AA-AGCTCAC -CCGTCCC-G AACACT---- GTCAGGAT-T GACAGAT--- TGAGAGCTCT

19 s ATGCGGCTT- AATTTGACTC AACAC--GGG AA-AGCTCAC -CCGTCCC-G AACACT---- GTCAGGAT-T GACAGAT--- TGAGAGCTCT

19 si ATGCGGCTT- AATTTGACTC AACAC--GGG AA-AGCTCAC -CCGTCCC-G AACACT---- GTCAGGAT-T GACAGAT--- TGAGAGCTCT

21 s ATGCGGCTT- AATTTGACTC AACAC--GGG AA-AGCTCAC -CCGTCCC-G AACACT---- GTCAGGAT-T GACAGAT--- TGAGAGCTCT

230 s ATGCGGCTT- AATTTGACTC AACAC--GGG AA-AGCTCAC -CCGTCCC-G AACACT---- GTCAGGAT-T GACAGAT--- TGAGAGCTCT

236 s ATGCGGCTT- AATTTGACTC AACAC--GGG AA-AGCTCAC -CCGTCCC-G AACACT---- GTCAGGAT-T GACAGAT--- TGAGAGCTCT

238 s ATGCGGCTT- AATTTGACTC AACAC--GGG AA-AGCTCAC -CCGTCCC-G AACACT---- GTCAGGAT-T GACAGAT--- TGAGAGCTCT

246 s ATGCGGCTT- AATTTGACTC AACAC--GGG AA-AGCTCAC -CCGTCCC-G AACACT---- GTCAGGAT-T GACAGAT--- TGAGAGCTCT

248 d ATGCGGCTT- AATTTGACTC AACAC--GGG AA-AGCTCAC -CCGTCCC-G AACACT---- GTCAGGAT-T GACAGAT--- TGAGAGCTCT

248 s ATGCGGCTT- AATTTGACTC AACAC--GGG AA-AGCTCAC -CCGTCCC-G AACACT---- GTCAGGAT-T GACAGAT--- TGAGAGCTCT

249 s ATGCGGCTT- AATTTGACTC AACAC--GGG AA-AGCTCAC -CCGTCCC-G AACACT---- GTCAGGAT-T GACAGAT--- TGAGAGCTCT

255 s ATGCGGCTT- AATTTGACTC AACAC--GGG AA-AGCTCAC -CCGTCCC-G AACACT---- GTCAGGAT-T GACAGAT--- TGAGAGCTCT

258 s ATGCGGCTT- AATTTGACTC AACAC--GGG AA-AGCTCAC -CCGTCCC-G AACACT---- GTCAGGAT-T GACAGAT--- TGAGAGCTCT

27 s ATGCGGCTT- AATTTGACTC AACAC--GGG AA-AGCTCAC -CCGTCCC-G AACACT---- GTCAGGAT-T GACAGAT--- TGAGAGCTCT

28 s ATGCGGCTT- AATTTGACTC AACAC--GGG AA-AGCTCAC -CCGTCCC-G AACACT---- GTCAGGAT-T GACAGAT--- TGAGAGCTCT

290 d ATGCGGCTT- AATTTGACTC AACAC--GGG AA-AGCTCAC -CCGTCCC-G AACACT---- GTCAGGAT-T GACAGAT--- TGAGAGCTCT

290 s ATGCGGCTT- AATTTGACTC AACAC--GGG AA-AGCTCAC -CCGTCCC-G AACACT---- GTCAGGAT-T GACAGAT--- TGAGAGCTCT

294 s ATGCGGCTT- AATTTGACTC AACAC--GGG AA-AGCTCAC -CCGTCCC-G AACACT---- GTCAGGAT-T GACAGAT--- TGAGAGCTCT

297 s ATGCGGCTT- AATTTGACTC AACAC--GGG AA-AGCTCAC -CCGTCCC-G AACACT---- GTCAGGAT-T GACAGAT--- TGAGAGCTCT

300 s ATGCGGCTT- AATTTGACTC AACAC--GGG AA-AGCTCAC -CCGTCCC-G AACACT---- GTCAGGAT-T GACAGAT--- TGAGAGCTCT

332 s ATGCGGCTT- AATTTGACTC AACAC--GGG AA-AGCTCAC -CCGTCCC-G AACACT---- GTCAGGAT-T GACAGAT--- TGAGAGCTCT

342 s ATGCGGCTT- AATTTGACTC AACAC--GGG AA-AGCTCAC -CCGTCCC-G AACACT---- GTCAGGAT-T GACAGAT--- TGAGAGCTCT

350 d ATGCGGCTT- AATTTGACTC AACAC--GGG AA-AGCTCAC -CCGTCCC-G AACACT---- GTCAGGAT-T GACAGAT--- TGAGAGCTCT

350 s ATGCGGCTT- AATTTGACTC AACAC--GGG AA-AGCTCAC -CCGTCCC-G AACACT---- GTCAGGAT-T GACAGAT--- TGAGAGCTCT

358 s ATGCGGCTT- AATTTGACTC AACAC--GGG AA-AGCTCAC -CCGTCCC-G AACACT---- GTCAGGAT-T GACAGAT--- TGAGAGCTCT

367 s ATGCGGCTT- AATTTGACTC AACAC--GGG AA-AGCTCAC -CCGTCCC-G AACACT---- GTCAGGAT-T GACAGAT--- TGAGAGCTCT

375 d ATGCGGCTT- AATTTGACTC AACAC--GGG AA-AGCTCAC -CCGTCCC-G AACACT---- GTCAGGAT-T GACAGAT--- TGAGAGCTCT

375 s ATGCGGCTT- AATTTGACTC AACAC--GGG AA-AGCTCAC -CCGTCCC-G AACACT---- GTCAGGAT-T GACAGAT--- TGAGAGCTCT

375 si ATGCGGCTT- AATTTGACTC AACAC--GGG AA-AGCTCAC -CCGTCCC-G AACACT---- GTCAGGAT-T GACAGAT--- TGAGAGCTCT

454 s ATGCGGCTT- AATTTGACTC AACAC--GGG AA-AGCTCAC -CCGTCCC-G AACACT---- GTCAGGAT-T GACAGAT--- TGAGAGCTCT

516 s ATGCGGCTT- AATTTGACTC AACAC--GGG AA-AGCTCAC -CCGTCCC-G AACACT---- GTCAGGAT-T GACAGAT--- TGAGAGCTCT

561 s ATGCGGCTT- AATTTGACTC AACAC--GGG AA-AGCTCAC -CCGTCCC-G AACACT---- GTCAGGAT-T GACAGAT--- TGAGAGCTCT

615 d ATGCGGCTT- AATTTGACTC AACAC--GGG AA-AGCTCAC -CCGTCCC-G AACACT---- GTCAGGAT-T GACAGAT--- TGAGAGCTCT

615 s ATGCGGCTT- AATTTGACTC AACAC--GGG AA-AGCTCAC -CCGTCCC-G AACACT---- GTCAGGAT-T GACAGAT--- TGAGAGCTCT

64 s ATGCGGCTT- AATTTGACTC AACAC--GGG AA-AGCTCAC -CCGTCCC-G AACACT---- GTCAGGAT-T GACAGAT--- TGAGAGCTCT

658 d ATGCGGCTT- AATTTGACTC AACAC--GGG AA-AGCTCAC -CCGTCCC-G AACACT---- GTCAGGAT-T GACAGAT--- TGAGAGCTCT

658 s ATGCGGCTT- AATTTGACTC AACAC--GGG AA-AGCTCAC -CCGTCCC-G AACACT---- GTCAGGAT-T GACAGAT--- TGAGAGCTCT

662 d ATGCGGCTT- AATTTGACTC AACAC--GGG AA-AGCTCAC -CCGTCCC-G AACACT---- GTCAGGAT-T GACAGAT--- TGAGAGCTCT

662 s ATGCGGCTT- AATTTGACTC AACAC--GGG AA-AGCTCAC -CCGTCCC-G AACACT---- GTCAGGAT-T GACAGAT--- TGAGAGCTCT

757 s ATGCGGCTT- AATTTGACTC AACAC--GGG AA-AGCTCAC -CCGTCCC-G AACACT---- GTCAGGAT-T GACAGAT--- TGAGAGCTCT

768 d ATGCGGCTT- AATTTGACTC AACAC--GGG AA-AGCTCAC -CCGTCCC-G AACACT---- GTCAGGAT-T GACAGAT--- TGAGAGCTCT

768 s ATGCGGCTT- AATTTGACTC AACAC--GGG AA-AGCTCAC -CCGTCCC-G AACACT---- GTCAGGAT-T GACAGAT--- TGAGAGCTCT

796 d ATGCGGCTT- AATTTGACTC AACAC--GGG AA-AGCTCAC -CCGTCCC-G AACACT---- GTCAGGAT-T GACAGAT--- TGAGAGCTCT

796 s ATGCGGCTT- AATTTGACTC AACAC--GGG AA-AGCTCAC -CCGTCCC-G AACACT---- GTCAGGAT-T GACAGAT--- TGAGAGCTCT

798 d ATGCGGCTT- AATTTGACTC AACAC--GGG AA-AGCTCAC -CCGTCCC-G AACACT---- GTCAGGAT-T GACAGAT--- TGAGAGCTCT

798 s ATGCGGCTT- AATTTGACTC AACAC--GGG AA-AGCTCAC -CCGTCCC-G AACACT---- GTCAGGAT-T GACAGAT--- TGAGAGCTCT

8 si ATGCGGCTT- AATTTGACTC AACAC--GGG AA-AGCTCAC -CCGTCCC-G AACACT---- GTCAGGAT-T GACAGAT--- TGAGAGCTCT

805 s ATGCGGCTT- AATTTGACTC AACAC--GGG AA-AGCTCAC -CCGTCCC-G AACACT---- GTCAGGAT-T GACAGAT--- TGAGAGCTCT

811 s ATGCGGCTT- AATTTGACTC AACAC--GGG AA-AGCTCAC -CCGTCCC-G AACACT---- GTCAGGAT-T GACAGAT--- TGAGAGCTCT

92 s ATGCGGCTT- AATTTGACTC AACAC--GGG AA-AGCTCAC -CCGTCCC-G AACACT---- GTCAGGAT-T GACAGAT--- TGAGAGCTCT

94 s ATGCGGCTT- AATTTGACTC AACAC--GGG AA-AGCTCAC -CCGTCCC-G AACACT---- GTCAGGAT-T GACAGAT--- TGAGAGCTCT

230 si CTGCGGCTT- AATTTGACTC AACAC--GGG AA-AACTCAC -CCGGCCC-G GACACC---- GCAAGGAT-T GACAGAT--- TGAAAGCTCT

454 d CTGCGGCTT- AATTTGACTC AACAC--GGG AA-AACTCAC -CCGGCCC-G GACACC---- GCAAGGAT-T GACAGAT--- TGAAAGCTCT

332 d CTGCGGCTT- AATTTGACTC AACAC--GGG AA-AACTCAC -CCGGCCC-G GACACC---- GCAAGGAT-T GACAGAT--- TGAAAGCTCT

454 s CTGCGGCTT- AATTTGACTC AACAC--GGG AA-AACTCAC -CCGGCCC-G GACACC---- GCAAGGAT-T GACAGAT--- TGAAAGCTCT

621 d CTGCGGCTT- AATTTGACTC AACAC--GGG AA-AACTCAC -CCGGCCC-G GACACC---- GCAAGGAT-T GACAGAT--- TGAAAGCTCT

64 si CTGCGGCTT- AATTTGACTC AACAC--GGG AA-AACTCAC -CCGGCCC-G GACACC---- GTAAGGAT-T GACAGAT--- TGAAAGCTCT

621 d male M. patens CTGCGGCTT- AATTTGACTC AACAC--GGG AA-AACTCAC -CCGGCCC-G GACACC---- GCAAGGAT-T GACAGAT--- TGAAAGCTCT

LC052360.1 A. putorii ATGCGGCTT- AATTTGACTC AACAC--GGG AA-AGCTCAC -CCGTCCC-G AACACT---- GTCAGGAT-T GACAGAT--- TGAGAGCTCT

JX456630.1 E. boehmi ATGCGGCTT- AATTTGACTC AACGC--GGG AA-AGTTCAC -CCGTCCC-G GACACT---- GTCAGGAT-T GACAGAT--- CAAGAGCTCT

KX962352.1 C. plica ATGCGGCTT- AATTTGACTC AACAC--GGG AA-AGCTCAC -CCGTCCC-G AACACT---- GTCAGGAT-T GACAGAT--- TGAGAGCTCT

MF287972.1 C. hepaticum ATGCGGCTT- AATTTGACTC AACAC--GGG AA-AGCTCAC -CCGTCCC-G AACACT---- GTCAGGAT-T GACAGAT--- TGAGAGCTCT

KC753538.1 C. splenaecum ATGCGGCTT- AATTTGACTC AACAC--GGG AA-AGCTCAC -CCGTCCC-G AACACT---- GTCAGGAT-T GACAGAT--- TGAGAGCTCT

KC341985.1 T. vulpis ATGCGGCTT- AATTTGACTC AACAC--GGG AA-AACTCAC -CCGTCCC-G AACACT---- GTGAGGAT-T GACAGAT--- CAAGAGCTCT

190 200 210 220 230 240 250 260 270

....|....| ....|....| ....|....| ....|....| ....|....| ....|....| ....|....| ....|....| ....|....|

131 s TTCTTGATTC AGTGGGT--- AGTGGTG--- ---------- CATGGCCGTT CTTAG----- -TTGGTGGA- GCGATTTGTC TGGCCAATCC

19 s TTCTTGATTC AGTGGGT--- AGTGGTG--- ---------- CATGGCCGTT CTTAG----- -TTGGTGGA- GCGATTTGTC TGGCCAATCC

19 si TTCTTGATTC AGTGGGT--- AGTGGTG--- ---------- CATGGCCGTT CTTAG----- -TTGGTGGA- GCGATTTGTC TGGCCAATCC

21 s TTCTTGATTC AGTGGGT--- AGTGGTG--- ---------- CATGGCCGTT CTTAG----- -TTGGTGGA- GCGATTTGTC TGGCCAATCC

230 s TTCTTGATTC AGTGGGT--- AGTGGTG--- ---------- CATGGCCGTT CTTAG----- -TTGGTGGA- GCGATTTGTC TGGCCAATCC

236 s TTCTTGATTC AGTGGGT--- AGTGGTG--- ---------- CATGGCCGTT CTTAG----- -TTGGTGGA- GCGATTTGTC TGGCCAATCC

238 s TTCTTGATTC AGTGGGT--- AGTGGTG--- ---------- CATGGCCGTT CTTAG----- -TTGGTGGA- GCGATTTGTC TGGCCAATCC

246 s TTCTTGATTC AGTGGGT--- AGTGGTG--- ---------- CATGGCCGTT CTTAG----- -TTGGTGGA- GCGATTTGTC TGGCCAATCC

248 d TTCTTGATTC AGTGGGT--- AGTGGTG--- ---------- CATGGCCGTT CTTAG----- -TTGGTGGA- GCGATTTGTC TGGCCAATCC

248 s TTCTTGATTC AGTGGGT--- AGTGGTG--- ---------- CATGGCCGTT CTTAG----- -TTGGTGGA- GCGATTTGTC TGGCCAATCC

249 s TTCTTGATTC AGTGGGT--- AGTGGTG--- ---------- CATGGCCGTT CTTAG----- -TTGGTGGA- GCGATTTGTC TGGCCAATCC

255 s TTCTTGATTC AGTGGGT--- AGTGGTG--- ---------- CATGGCCGTT CTTAG----- -TTGGTGGA- GCGATTTGTC TGGCCAATCC

258 s TTCTTGATTC AGTGGGT--- AGTGGTG--- ---------- CATGGCCGTT CTTAG----- -TTGGTGGA- GCGATTTGTC TGGCCAATCC

27 s TTCTTGATTC AGTGGGT--- AGTGGTG--- ---------- CATGGCCGTT CTTAG----- -TTGGTGGA- GCGATTTGTC TGGCCAATCC

28 s TTCTTGATTC AGTGGGT--- AGTGGTG--- ---------- CATGGCCGTT CTTAG----- -TTGGTGGA- GCGATTTGTC TGGCCAATCC

290 d TTCTTGATTC AGTGGGT--- AGTGGTG--- ---------- CATGGCCGTT CTTAG----- -TTGGTGGA- GCGATTTGTC TGGCCAATCC

290 s TTCTTGATTC AGTGGGT--- AGTGGTG--- ---------- CATGGCCGTT CTTAG----- -TTGGTGGA- GCGATTTGTC TGGCCAATCC

294 s TTCTTGATTC AGTGGGT--- AGTGGTG--- ---------- CATGGCCGTT CTTAG----- -TTGGTGGA- GCGATTTGTC TGGCCAATCC

297 s TTCTTGATTC AGTGGGT--- AGTGGTG--- ---------- CATGGCCGTT CTTAG----- -TTGGTGGA- GCGATTTGTC TGGCCAATCC

300 s TTCTTGATTC AGTGGGT--- AGTGGTG--- ---------- CATGGCCGTT CTTAG----- -TTGGTGGA- GCGATTTGTC TGGCCAATCC

332 s TTCTTGATTC AGTGGGT--- AGTGGTG--- ---------- CATGGCCGTT CTTAG----- -TTGGTGGA- GCGATTTGTC TGGCCAATCC

342 s TTCTTGATTC AGTGGGT--- AGTGGTG--- ---------- CATGGCCGTT CTTAG----- -TTGGTGGA- GCGATTTGTC TGGCCAATCC

350 d TTCTTGATTC AGTGGGT--- AGTGGTG--- ---------- CATGGCCGTT CTTAG----- -TTGGTGGA- GCGATTTGTC TGGCCAATCC

350 s TTCTTGATTC AGTGGGT--- AGTGGTG--- ---------- CATGGCCGTT CTTAG----- -TTGGTGGA- GCGATTTGTC TGGCCAATCC

358 s TTCTTGATTC AGTGGGT--- AGTGGTG--- ---------- CATGGCCGTT CTTAG----- -TTGGTGGA- GCGATTTGTC TGGCCAATCC

367 s TTCTTGATTC AGTGGGT--- AGTGGTG--- ---------- CATGGCCGTT CTTAG----- -TTGGTGGA- GCGATTTGTC TGGCCAATCC

375 d TTCTTGATTC AGTGGGT--- AGTGGTG--- ---------- CATGGCCGTT CTTAG----- -TTGGTGGA- GCGATTTGTC TGGCCAATCC

375 s TTCTTGATTC AGTGGGT--- AGTGGTG--- ---------- CATGGCCGTT CTTAG----- -TTGGTGGA- GCGATTTGTC TGGCCAATCC

375 si TTCTTGATTC AGTGGGT--- AGTGGTG--- ---------- CATGGCCGTT CTTAG----- -TTGGTGGA- GCGATTTGTC TGGCCAATCC

454 s TTCTTGATTC AGTGGGT--- AGTGGTG--- ---------- CATGGCCGTT CTTAG----- -TTGGTGGA- GCGATTTGTC TGGCCAATCC

516 s TTCTTGATTC AGTGGGT--- AGTGGTG--- ---------- CATGGCCGTT CTTAG----- -TTGGTGGA- GCGATTTGTC TGGCCAATCC

561 s TTCTTGATTC AGTGGGT--- AGTGGTG--- ---------- CATGGCCGTT CTTAG----- -TTGGTGGA- GCGATTTGTC TGGCCAATCC

615 d TTCTTGATTC AGTGGGT--- AGTGGTG--- ---------- CATGGCCGTT CTTAG----- -TTGGTGGA- GCGATTTGTC TGGCCAATCC

615 s TTCTTGATTC AGTGGGT--- AGTGGTG--- ---------- CATGGCCGTT CTTAG----- -TTGGTGGA- GCGATTTGTC TGGCCAATCC

64 s TTCTTGATTC AGTGGGT--- AGTGGTG--- ---------- CATGGCCGTT CTTAG----- -TTGGTGGA- GCGATTTGTC TGGCCAATCC

658 d TTCTTGATTC AGTGGGT--- AGTGGTG--- ---------- CATGGCCGTT CTTAG----- -TTGGTGGA- GCGATTTGTC TGGCCAATCC

658 s TTCTTGATTC AGTGGGT--- AGTGGTG--- ---------- CATGGCCGTT CTTAG----- -TTGGTGGA- GCGATTTGTC TGGCCAATCC

662 d TTCTTGATTC AGTGGGT--- AGTGGTG--- ---------- CATGGCCGTT CTTAG----- -TTGGTGGA- GCGATTTGTC TGGCCAATCC

662 s TTCTTGATTC AGTGGGT--- AGTGGTG--- ---------- CATGGCCGTT CTTAG----- -TTGGTGGA- GCGATTTGTC TGGCCAATCC

757 s TTCTTGATTC AGTGGGT--- AGTGGTG--- ---------- CATGGCCGTT CTTAG----- -TTGGTGGA- GCGATTTGTC TGGCCAATCC

768 d TTCTTGATTC AGTGGGT--- AGTGGTG--- ---------- CATGGCCGTT CTTAG----- -TTGGTGGA- GCGATTTGTC TGGCCAATCC

768 s TTCTTGATTC AGTGGGT--- AGTGGTG--- ---------- CATGGCCGTT CTTAG----- -TTGGTGGA- GCGATTTGTC TGGCCAATCC

796 d TTCTTGATTC AGTGGGT--- AGTGGTG--- ---------- CATGGCCGTT CTTAG----- -TTGGTGGA- GCGATTTGTC TGGCCAATCC

796 s TTCTTGATTC AGTGGGT--- AGTGGTG--- ---------- CATGGCCGTT CTTAG----- -TTGGTGGA- GCGATTTGTC TGGCCAATCC

798 d TTCTTGATTC AGTGGGT--- AGTGGTG--- ---------- CATGGCCGTT CTTAG----- -TTGGTGGA- GCGATTTGTC TGGCCAATCC

798 s TTCTTGATTC AGTGGGT--- AGTGGTG--- ---------- CATGGCCGTT CTTAG----- -TTGGTGGA- GCGATTTGTC TGGCCAATCC

8 si TTCTTGATTC AGTGGGT--- AGTGGTG--- ---------- CATGGCCGTT CTTAG----- -TTGGTGGA- GCGATTTGTC TGGCCAATCC

805 s TTCTTGATTC AGTGGGT--- AGTGGTG--- ---------- CATGGCCGTT CTTAG----- -TTGGTGGA- GCGATTTGTC TGGCCAATCC

811 s TTCTTGATTC AGTGGGT--- AGTGGTG--- ---------- CATGGCCGTT CTTAG----- -TTGGTGGA- GCGATTTGTC TGGCCAATCC

92 s TTCTTGATTC AGTGGGT--- AGTGGTG--- ---------- CATGGCCGTT CTTAG----- -TTGGTGGA- GCGATTTGTC TGGCCAATCC

94 s TTCTTGATTC AGTGGGT--- AGTGGTG--- ---------- CATGGCCGTT CTTAG----- -TTGGTGGA- GCGATTTGTC TGGCCAATCC

230 si TTCTCGATTT GGTGGTT--- GGTGGTG--- ---------- CATGGCCGTT CTTAG----- -TTGGTGGA- GCGATTTGTC TGGTTTATTC

454 d TTCTCGATTT GGTGGTT--- GGTGGTG--- ---------- CATGGCCGTT CTTAG----- -TTGGTGGA- GCGATTTGTC TGGTTTATTC

332 d TTCTCGATTT GGTGGTT--- GGTGGTG--- ---------- CATGGCCGTT CTTAG----- -TTGGTGGA- GCGATTTGTC TGGTTTATTC

454 s TTCTCGATTT GGTGGTT--- GGTGGTG--- ---------- CATGGCCGTT CTTAG----- -TTGGTGGA- GCGATTTGTC TGGTTTATTC

621 d TTCTCGATTT GGTGGTT--- GGTGGTG--- ---------- CATGGCCGTT CTTAG----- -TTGGTGGA- GCGATTTGTC TGGTTTATTC

64 si TTCTCGATTT GGTGGTT--- GGTGGTG--- ---------- CATGGCCGTT CTTAG----- -TTGGTGGA- GCGATTTGTC TGGTTTATTC

621 d male M. patens TTCTCGATTT GGTGGTT--- GGTGGTG--- ---------- CATGGCCGTT CTTAG----- -TTGGTGGA- GCGATTTGTC TGGTTTATTC

LC052360.1 A. putorii TTCTTGATTC AGTGGGT--- AGTGGTG--- ---------- CATGGCCGTT CTTAGT---- --TGGTGGA- GCGATTTGTC TGGCCAATCC

JX456630.1 E. boehmi TTCTTGATTC AGTGGAT--- AGTGGTG--- ---------- CATGGCCGTT CTTAGT---- --TGGTGGA- GCGATTTGTC TGGCCAATCC

KX962352.1 C. plica TTCTTGATTC AGTGGGT--- AGTGGTG--- ---------- CATGGCCGTT CTTAGT---- --TGGTGGA- GCGATTTGTC TGGCCAATCC

MF287972.1 C. hepaticum TTCTTGATTC AGTGGGT--- AGTGGTG--- ---------- CATGGCCGTT CTTAGT---- --TGGTGGA- GCGATTTGTC TGGCCAATCC

KC753538.1 C. splenaecum TTCTTGATTC AGTGGGT--- AGTGGTG--- ---------- CATGGCCGTT CTTAGT---- --TGGTGGA- GCGATTTGTC TGGCCGATCC

KC341985.1 T. vulpis TTCTTGATTC AGTGGGT--- AGTGGTG--- ---------- CATGGCCGTT CTTAGT---- --TGGTGGA- GCGATTTGTC TGGCTAATTC

280 290 300 310 320 330 340 350 360

....|....| ....|....| ....|....| ....|....| ....|....| ....|....| ....|....| ....|....| ....|....|

131 s CGATA--ACG AACGAGACTC ------TGGC C--TACTAAA -TAGTGACG- ----GC-TC- -----AATTT TTGCT----- ----------

19 s CGATA--ACG AACGAGACTC ------TGGC C--TACTAAA -TAGTGACG- ----GC-TC- -----AATTT TTGCT----- ----------

19 si CGATA--ACG AACGAGACTC ------TGGC C--TACTAAA -TAGTGACG- ----GC-TC- -----AATTT TTGCT----- ----------

21 s CGATA--ACG AACGAGACTC ------TGGC C--TACTAAA -TAGTGACG- ----GC-TC- -----AATTT TTGCT----- ----------

230 s CGATA--ACG AACGAGACTC ------TGGC C--TACTAAA -TAGTGACG- ----GC-TC- -----AATTT TTGCT----- ----------

236 s CGATA--ACG AACGAGACTC ------TGGC C--TACTAAA -TAGTGACG- ----GC-TC- -----AATTT TTGCT----- ----------

238 s CGATA--ACG AACGAGACTC ------TGGC C--TACTAAA -TAGTGACG- ----GC-TC- -----AATTT TTGCT----- ----------

246 s CGATA--ACG AACGAGACTC ------TGGC C--TACTAAA -TAGTGACG- ----GC-TC- -----AATTT TTGCT----- ----------

248 d CGATA--ACG AACGAGACTC ------TGGC C--TACTAAA -TAGTGACG- ----GC-TC- -----AATTT TTGCT----- ----------

248 s CGATA--ACG AACGAGACTC ------TGGC C--TACTAAA -TAGTGACG- ----GC-TC- -----AATTT TTGCT----- ----------

249 s CGATA--ACG AACGAGACTC ------TGGC C--TACTAAA -TAGTGACG- ----GC-TC- -----AATTT TTGCT----- ----------

255 s CGATA--ACG AACGAGACTC ------TGGC C--TACTAAA -TAGTGACG- ----GC-TC- -----AATTT TTGCT----- ----------

258 s CGATA--ACG AACGAGACTC ------TGGC C--TACTAAA -TAGTGACG- ----GC-TC- -----AATTT TTGCT----- ----------

27 s CGATA--ACG AACGAGACTC ------TGGC C--TACTAAA -TAGTGACG- ----GC-TC- -----AATTT TTGCT----- ----------

28 s CGATA--ACG AACGAGACTC ------TGGC C--TACTAAA -TAGTGACG- ----GC-TC- -----AATTT TTGCT----- ----------

290 d CGATA--ACG AACGAGACTC ------TGGC C--TACTAAA -TAGTGACG- ----GC-TC- -----AATTT TTGCT----- ----------

290 s CGATA--ACG AACGAGACTC ------TGGC C--TACTAAA -TAGTGACG- ----GC-TC- -----AATTT TTGCT----- ----------

294 s CGATA--ACG AACGAGACTC ------TGGC C--TACTAAA -TAGTGACG- ----GC-TC- -----AATTT TTGCT----- ----------

297 s CGATA--ACG AACGAGACTC ------TGGC C--TACTAAA -TAGTGACG- ----GC-TC- -----AATTT TTGCT----- ----------

300 s CGATA--ACG AACGAGACTC ------TGGC C--TACTAAA -TAGTGACG- ----GC-TC- -----AATTT TTGCT----- ----------

332 s CGATA--ACG AACGAGACTC ------TGGC C--TACTAAA -TAGTGACG- ----GC-TC- -----AATTT TTGCT----- ----------

342 s CGATA--ACG AACGAGACTC ------TGGC C--TACTAAA -TAGTGACG- ----GC-TC- -----AATTT TTGCT----- ----------

350 d CGATA--ACG AACGAGACTC ------TGGC C--TACTAAA -TAGTGACG- ----GC-TC- -----AATTT TTGCT----- ----------

350 s CGATA--ACG AACGAGACTC ------TGGC C--TACTAAA -TAGTGACG- ----GC-TC- -----AATTT TTGCT----- ----------

358 s CGATA--ACG AACGAGACTC ------TGGC C--TACTAAA -TAGTGACG- ----GC-TC- -----AATTT TTGCT----- ----------

367 s CGATA--ACG AACGAGACTC ------TGGC C--TACTAAA -TAGTGACG- ----GC-TC- -----AATTT TTGCT----- ----------

375 d CGATA--ACG AACGAGACTC ------TGGC C--TACTAAA -TAGTGACG- ----GC-TC- -----AATTT TTGCT----- ----------

375 s CGATA--ACG AACGAGACTC ------TGGC C--TACTAAA -TAGTGACG- ----GC-TC- -----AATTT TTGCT----- ----------

375 si CGATA--ACG AACGAGACTC ------TGGC C--TACTAAA -TAGTGACG- ----GC-TC- -----AATTT TTGCT----- ----------

454 s CGATA--ACG AACGAGACTC ------TGGC C--TACTAAA -TAGTGACG- ----GC-TC- -----AATTT TTGCT----- ----------

516 s CGATA--ACG AACGAGACTC ------TGGC C--TACTAAA -TAGTGACG- ----GC-TC- -----AATTT TTGCT----- ----------

561 s CGATA--ACG AACGAGACTC ------TGGC C--TACTAAA -TAGTGACG- ----GC-TC- -----AATTT TTGCT----- ----------

615 d CGATA--ACG AACGAGACTC ------TGGC C--TACTAAA -TAGTGACG- ----GC-TC- -----AATTT TTGCT----- ----------

615 s CGATA--ACG AACGAGACTC ------TGGC C--TACTAAA -TAGTGACG- ----GC-TC- -----AATTT TTGCT----- ----------

64 s CGATA--ACG AACGAGACTC ------TGGC C--TACTAAA -TAGTGACG- ----GC-TC- -----AATTT TTGCT----- ----------

658 d CGATA--ACG AACGAGACTC ------TGGC C--TACTAAA -TAGTGACG- ----GC-TC- -----AATTT TTGCT----- ----------

658 s CGATA--ACG AACGAGACTC ------TGGC C--TACTAAA -TAGTGACG- ----GC-TC- -----AATTT TTGCT----- ----------

662 d CGATA--ACG AACGAGACTC ------TGGC C--TACTAAA -TAGTGACG- ----GC-TC- -----AATTT TTGCT----- ----------

662 s CGATA--ACG AACGAGACTC ------TGGC C--TACTAAA -TAGTGACG- ----GC-TC- -----AATTT TTGCT----- ----------

757 s CGATA--ACG AACGAGACTC ------TGGC C--TACTAAA -TAGTGACG- ----GC-TC- -----AATTT TTGCT----- ----------

768 d CGATA--ACG AACGAGACTC ------TGGC C--TACTAAA -TAGTGACG- ----GC-TC- -----AATTT TTGCT----- ----------

768 s CGATA--ACG AACGAGACTC ------TGGC C--TACTAAA -TAGTGACG- ----GC-TC- -----AATTT TTGCT----- ----------

796 d CGATA--ACG AACGAGACTC ------TGGC C--TACTAAA -TAGTGACG- ----GC-TC- -----AATTT TTGCT----- ----------

796 s CGATA--ACG AACGAGACTC ------TGGC C--TACTAAA -TAGTGACG- ----GC-TC- -----AATTT TTGCT----- ----------

798 d CGATA--ACG AACGAGACTC ------TGGC C--TACTAAA -TAGTGACG- ----GC-TC- -----AATTT TTGCT----- ----------

798 s CGATA--ACG AACGAGACTC ------TGGC C--TACTAAA -TAGTGACG- ----GC-TC- -----AATTT TTGCT----- ----------

8 si CGATA--ACG AACGAGACTC ------TGGC C--TACTAAA -TAGTGACG- ----GC-TC- -----AATTT TTGCT----- ----------

805 s CGATA--ACG AACGAGACTC ------TGGC C--TACTAAA -TAGTGACG- ----GC-TC- -----AATTT TTGCT----- ----------

811 s CGATA--ACG AACGAGACTC ------TGGC C--TACTAAA -TAGTGACG- ----GC-TC- -----AATTT TTGCT----- ----------

92 s CGATA--ACG AACGAGACTC ------TGGC C--TACTAAA -TAGTGACG- ----GC-TC- -----AATTT TTGCT----- ----------

94 s CGATA--ACG AACGAGACTC ------TGGC C--TACTAAA -TAGTGACG- ----GC-TC- -----AATTT TTGCT----- ----------

230 si CGATA--ACG AGCGAGACTC ------TAGC C--TGCTAAA -TAGTGGCT- ----G----- -----GATTT TTAAG----- ----------

454 d CGATA--ACG AGCGAGACTC ------TAGC C--TGCTAAA -TAGTGGCT- ----G----- -----GATTT TTAAG----- ----------

332 d CGATA--ACG AGCGAGACTC ------TAGC C--TGCTAAA -TAGTGGCT- ----G----- -----GATTT TTAAG----- ----------

454 s CGATA--ACG AGCGAGACTC ------TAGC C--TGCTAAA -TAGTGGCT- ----G----- -----GATTT TTAAG----- ----------

621 d CGATA--ACG AGCGAGACTC ------TAGC C--TGCTAAA -TAGTGGCT- ----G----- -----GATTT TTAAG----- ----------

64 si CGATA--ACG AGCGAGACTC ------TAGC C--TGCTAAA -TAGTGACT- ----A----- -----GATTT TTATG----- ----------

621 d male M. patens CGATA--ACG AGCGAGACTC ------TAGC C--TGCTAAA -TAGTGGCT- ----G----- -----GATTT TTAAG----- ----------

LC052360.1 A. putorii CGATA--ACG AACGAGACTC ------TGGC C--TACTAAA -TAGTGACG- ----GC-TCA ------A--T TTGCT----- ----------

JX456630.1 E. boehmi CGATA--ACG AACGAGACTC ------TGGC C--TACTAAC -TAGTGACG- ----GCGTG- ------GTCT TTT------- ----------

KX962352.1 C. plica CGATA--ACG AACGAGACTC ------TGGC C--TACTAAA -TAGTGACG- ----GC-TCA ------A--T TTGCT----- ----------

MF287972.1 C. hepaticum CGATA--ACG AACGAGACTC ------TGGC C--TACTAAA -TAGTGACG- ----GC-TCA ------A--T TTGCT----- ----------

KC753538.1 C. splenaecum CGATA--ACG AACGAGACTC ------TGGC T--TACTAAA -TAGTGACG- ----GC-TCA ------A--T TTGCT----- ----------

KC341985.1 T. vulpis CGATA--ACG AACGAGACTC ------TGGC C--TACTAAC -TAGCGGCG- ----GTGTTC ------ATGC CTCCTGACGG GGGCCGCGTG

370 380 390 400 410 420 430 440 450

....|....| ....|....| ....|....| ....|....| ....|....| ....|....| ....|....| ....|....| ....|....|

131 s -------TTG CCGAGCAC-- ---------- ---------- ---------- --TTCTT--A GAG-GGACAA ---GCGGCGG TTCA----AA

19 s -------TTG CCGAGCAC-- ---------- ---------- ---------- --TTCTT--A GAG-GGACAA ---GCGGCGG TTCA----AA

19 si -------TTG CCGAGCAC-- ---------- ---------- ---------- --TTCTT--A GAG-GGACAA ---GCGGCGG TTCA----AA

21 s -------TTG CCGAGCAC-- ---------- ---------- ---------- --TTCTT--A GAG-GGACAA ---GCGGCGG TTCA----AA

230 s -------TTG CCGAGCAC-- ---------- ---------- ---------- --TTCTT--A GAG-GGACAA ---GCGGCGG TTCA----AA

236 s -------TTG CCGAGCAC-- ---------- ---------- ---------- --TTCTT--A GAG-GGACAA ---GCGGCGG TTCA----AA

238 s -------TTG CCGAGCAC-- ---------- ---------- ---------- --TTCTT--A GAG-GGACAA ---GCGGCGG TTCA----AA

246 s -------TTG CCGAGCAC-- ---------- ---------- ---------- --TTCTT--A GAG-GGACAA ---GCGGCGG TTCA----AA

248 d -------TTG CCGAGCAC-- ---------- ---------- ---------- --TTCTT--A GAG-GGACAA ---GCGGCGG TTCA----AA

248 s -------TTG CCGAGCAC-- ---------- ---------- ---------- --TTCTT--A GAG-GGACAA ---GCGGCGG TTCA----AA

249 s -------TTG CCGAGCAC-- ---------- ---------- ---------- --TTCTT--A GAG-GGACAA ---GCGGCGG TTCA----AA

255 s -------TTG CCGAGCAC-- ---------- ---------- ---------- --TTCTT--A GAG-GGACAA ---GCGGCGG TTCA----AA

258 s -------TTG CCGAGCAC-- ---------- ---------- ---------- --TTCTT--A GAG-GGACAA ---GCGGCGG TTCA----AA

27 s -------TTG CCGAGCAC-- ---------- ---------- ---------- --TTCTT--A GAG-GGACAA ---GCGGCGG TTCA----AA

28 s -------TTG CCGAGCAC-- ---------- ---------- ---------- --TTCTT--A GAG-GGACAA ---GCGGCGG TTCA----AA

290 d -------TTG CCGAGCAC-- ---------- ---------- ---------- --TTCTT--A GAG-GGACAA ---GCGGCGG TTCA----AA

290 s -------TTG CCGAGCAC-- ---------- ---------- ---------- --TTCTT--A GAG-GGACAA ---GCGGCGG TTCA----AA

294 s -------TTG CCGAGCAC-- ---------- ---------- ---------- --TTCTT--A GAG-GGACAA ---GCGGCGG TTCA----AA

297 s -------TTG CCGAGCAC-- ---------- ---------- ---------- --TTCTT--A GAG-GGACAA ---GCGGCGG TTCA----AA

300 s -------TTG CCGAGCAC-- ---------- ---------- ---------- --TTCTT--A GAG-GGACAA ---GCGGCGG TTCA----AA

332 s -------TTG CCGAGCAC-- ---------- ---------- ---------- --TTCTT--A GAG-GGACAA ---GCGGCGG TTCA----AA

342 s -------TTG CCGAGCAC-- ---------- ---------- ---------- --TTCTT--A GAG-GGACAA ---GCGGCGG TTCA----AA

350 d -------TTG CCGAGCAC-- ---------- ---------- ---------- --TTCTT--A GAG-GGACAA ---GCGGCGG TTCA----AA

350 s -------TTG CCGAGCAC-- ---------- ---------- ---------- --TTCTT--A GAG-GGACAA ---GCGGCGG TTCA----AA

358 s -------TTG CCGAGCAC-- ---------- ---------- ---------- --TTCTT--A GAG-GGACAA ---GCGGCGG TTCA----AA

367 s -------TTG CCGAGCAC-- ---------- ---------- ---------- --TTCTT--A GAG-GGACAA ---GCGGCGG TTCA----AA

375 d -------TTG CCGAGCAC-- ---------- ---------- ---------- --TTCTT--A GAG-GGACAA ---GCGGCGG TTCA----AA

375 s -------TTG CCGAGCAC-- ---------- ---------- ---------- --TTCTT--A GAG-GGACAA ---GCGGCGG TTCA----AA

375 si -------TTG CCGAGCAC-- ---------- ---------- ---------- --TTCTT--A GAG-GGACAA ---GCGGCGG TTCA----AA

454 s -------TTG CCGAGCAC-- ---------- ---------- ---------- --TTCTT--A GAG-GGACAA ---GCGGCGG TTCA----AA

516 s -------TTG CCGAGCAC-- ---------- ---------- ---------- --TTCTT--A GAG-GGACAA ---GCGGCGG TTCA----AA

561 s -------TTG CCGAGCAC-- ---------- ---------- ---------- --TTCTT--A GAG-GGACAA ---GCGGCGG TTCA----AA

615 d -------TTG CCGAGCAC-- ---------- ---------- ---------- --TTCTT--A GAG-GGACAA ---GCGGCGG TTCA----AA

615 s -------TTG CCGAGCAC-- ---------- ---------- ---------- --TTCTT--A GAG-GGACAA ---GCGGCGG TTCA----AA

64 s -------TTG CCGAGCAC-- ---------- ---------- ---------- --TTCTT--A GAG-GGACAA ---GCGGCGG TTCA----AA

658 d -------TTG CCGAGCAC-- ---------- ---------- ---------- --TTCTT--A GAG-GGACAA ---GCGGCGG TTCA----AA

658 s -------TTG CCGAGCAC-- ---------- ---------- ---------- --TTCTT--A GAG-GGACAA ---GCGGCGG TTCA----AA

662 d -------TTG CCGAGCAC-- ---------- ---------- ---------- --TTCTT--A GAG-GGACAA ---GCGGCGG TTCA----AA

662 s -------TTG CCGAGCAC-- ---------- ---------- ---------- --TTCTT--A GAG-GGACAA ---GCGGCGG TTCA----AA

757 s -------TTG CCGAGCAC-- ---------- ---------- ---------- --TTCTT--A GAG-GGACAA ---GCGGCGG TTCA----AA

768 d -------TTG CCGAGCAC-- ---------- ---------- ---------- --TTCTT--A GAG-GGACAA ---GCGGCGG TTCA----AA

768 s -------TTG CCGAGCAC-- ---------- ---------- ---------- --TTCTT--A GAG-GGACAA ---GCGGCGG TTCA----AA

796 d -------TTG CCGAGCAC-- ---------- ---------- ---------- --TTCTT--A GAG-GGACAA ---GCGGCGG TTCA----AA

796 s -------TTG CCGAGCAC-- ---------- ---------- ---------- --TTCTT--A GAG-GGACAA ---GCGGCGG TTCA----AA

798 d -------TTG CCGAGCAC-- ---------- ---------- ---------- --TTCTT--A GAG-GGACAA ---GCGGCGG TTCA----AA

798 s -------TTG CCGAGCAC-- ---------- ---------- ---------- --TTCTT--A GAG-GGACAA ---GCGGCGG TTCA----AA

8 si -------TTG CCGAGCAC-- ---------- ---------- ---------- --TTCTT--A GAG-GGACAA ---GCGGCGG TTCA----AA

805 s -------TTG CCGAGCAC-- ---------- ---------- ---------- --TTCTT--A GAG-GGACAA ---GCGGCGG TTCA----AA

811 s -------TTG CCGAGCAC-- ---------- ---------- ---------- --TTCTT--A GAG-GGACAA ---GCGGCGG TTCA----AA

92 s -------TTG CCGAGCAC-- ---------- ---------- ---------- --TTCTT--A GAG-GGACAA ---GCGGCGG TTCA----AA

94 s -------TTG CCGAGCAC-- ---------- ---------- ---------- --TTCTT--A GAG-GGACAA ---GCGGCGG TTCA----AA

230 si -------TC- CAGTCTAC-- ---------- ---------- ---------- --TTCTT--A GAG-GGATAA ---GCGGTGT TT-------A

454 d -------TC- CAGTCTAC-- ---------- ---------- ---------- --TTCTT--A GAG-GGATAA ---GCGGTGT TT-------A

332 d -------TC- CAGTCTAC-- ---------- ---------- ---------- --TTCTT--A GAG-GGATAA ---GCGGTGT TT-------A

454 s -------TC- CAGTCTAC-- ---------- ---------- ---------- --TTCTT--A GAG-GGATAA ---GCGGTGT TT-------A

621 d -------TC- CAGTCTAC-- ---------- ---------- ---------- --TTCTT--A GAG-GGATAA ---GCGGTGT TT-------A

64 si -------TC- TAGTCTAC-- ---------- ---------- ---------- --TTCTT--A GAG-GGATAA ---GCGGTGT TT-------A

621 d male M. patens -------TC- CAGTCTAC-- ---------- ---------- ---------- --TTCTT--A GAG-GGATAA ---GCGGTGT TT-------A

LC052360.1 A. putorii -------TTG CCGAGCAC-- ---------- ---------- ---------- --TTCTT--A GAG-GGACAA ---GCGGCGG TACA----AA

JX456630.1 E. boehmi --------CG CTGAGCAC-- ---------- ---------- ---------- --TTCTT--A GAG-GGACCA ---GCGGCGG CACA----AA

KX962352.1 C. plica -------TTG CCGAGCAC-- ---------- ---------- ---------- --TTCTT--A GAG-GGACAA ---GCGGCGG TACA----AA

MF287972.1 C. hepaticum -------TTG CCGAGCAC-- ---------- ---------- ---------- --TTCTT--A GAG-GGACAA ---GCGGCGG TACA----AA

KC753538.1 C. splenaecum -------TTG CCGAGCAC-- ---------- ---------- ---------- --TTCTT--A GAG-GGACAA ---GCGGCAG TACA----AA

KC341985.1 T. vulpis CGGC-AACCG CCGGGCGCGC CCCTTGGAGC AGCAGCGCCG GCAGCCGC-- --TTCTT--A GAG-GGACCA ---GCGACAC TTTCGC--AA

460 470 480 490 500 510 520 530 540

....|....| ....|....| ....|....| ....|....| ....|....| ....|....| ....|....| ....|....| ....|....|

131 s GCC-GCAC-- -G----AGAA A-GAG-CAAT AA---CAGGT CTG--TG-AT GCCCTTA--- -GA--TGGAC G----GGGCT GC-ACG--CG

19 s GCC-GCAC-- -G----AGAA A-GAG-CAAT AA---CAGGT CTG--TG-AT GCCCTTA--- -GA--TGGAC G----GGGCT GC-ACG--CG

19 si GCC-GCAC-- -G----AGAA A-GAG-CAAT AA---CAGGT CTG--TG-AT GCCCTTA--- -GA--TGGAC G----GGGCT GC-ACG--CG

21 s GCC-GCAC-- -G----AGAA A-GAG-CAAT AA---CAGGT CTG--TG-AT GCCCTTA--- -GA--TGGAC G----GGGCT GC-ACG--CG

230 s GCC-GCAC-- -G----AGAA A-GAG-CAAT AA---CAGGT CTG--TG-AT GCCCTTA--- -GA--TGGAC G----GGGCT GC-ACG--CG

236 s GCC-GCAC-- -G----AGAA A-GAG-CAAT AA---CAGGT CTG--TG-AT GCCCTTA--- -GA--TGGAC G----GGGCT GC-ACG--CG

238 s GCC-GCAC-- -G----AGAA A-GAG-CAAT AA---CAGGT CTG--TG-AT GCCCTTA--- -GA--TGGAC G----GGGCT GC-ACG--CG

246 s GCC-GCAC-- -G----AGAA A-GAG-CAAT AA---CAGGT CTG--TG-AT GCCCTTA--- -GA--TGGAC G----GGGCT GC-ACG--CG

248 d GCC-GCAC-- -G----AGAA A-GAG-CAAT AA---CAGGT CTG--TG-AT GCCCTTA--- -GA--TGGAC G----GGGCT GC-ACG--CG

248 s GCC-GCAC-- -G----AGAA A-GAG-CAAT AA---CAGGT CTG--TG-AT GCCCTTA--- -GA--TGGAC G----GGGCT GC-ACG--CG

249 s GCC-GCAC-- -G----AGAA A-GAG-CAAT AA---CAGGT CTG--TG-AT GCCCTTA--- -GA--TGGAC G----GGGCT GC-ACG--CG

255 s GCC-GCAC-- -G----AGAA A-GAG-CAAT AA---CAGGT CTG--TG-AT GCCCTTA--- -GA--TGGAC G----GGGCT GC-ACG--CG

258 s GCC-GCAC-- -G----AGAA A-GAG-CAAT AA---CAGGT CTG--TG-AT GCCCTTA--- -GA--TGGAC G----GGGCT GC-ACG--CG

27 s GCC-GCAC-- -G----AGAA A-GAG-CAAT AA---CAGGT CTG--TG-AT GCCCTTA--- -GA--TGGAC G----GGGCT GC-ACG--CG

28 s GCC-GCAC-- -G----AGAA A-GAG-CAAT AA---CAGGT CTG--TG-AT GCCCTTA--- -GA--TGGAC G----GGGCT GC-ACG--CG

290 d GCC-GCAC-- -G----AGAA A-GAG-CAAT AA---CAGGT CTG--TG-AT GCCCTTA--- -GA--TGGAC G----GGGCT GC-ACG--CG

290 s GCC-GCAC-- -G----AGAA A-GAG-CAAT AA---CAGGT CTG--TG-AT GCCCTTA--- -GA--TGGAC G----GGGCT GC-ACG--CG

294 s GCC-GCAC-- -G----AGAA A-GAG-CAAT AA---CAGGT CTG--TG-AT GCCCTTA--- -GA--TGGAC G----GGGCT GC-ACG--CG

297 s GCC-GCAC-- -G----AGAA A-GAG-CAAT AA---CAGGT CTG--TG-AT GCCCTTA--- -GA--TGGAC G----GGGCT GC-ACG--CG

300 s GCC-GCAC-- -G----AGAA A-GAG-CAAT AA---CAGGT CTG--TG-AT GCCCTTA--- -GA--TGGAC G----GGGCT GC-ACG--CG

332 s GCC-GCAC-- -G----AGAA A-GAG-CAAT AA---CAGGT CTG--TG-AT GCCCTTA--- -GA--TGGAC G----GGGCT GC-ACG--CG

342 s GCC-GCAC-- -G----AGAA A-GAG-CAAT AA---CAGGT CTG--TG-AT GCCCTTA--- -GA--TGGAC G----GGGCT GC-ACG--CG

350 d GCC-GCAC-- -G----AGAA A-GAG-CAAT AA---CAGGT CTG--TG-AT GCCCTTA--- -GA--TGGAC G----GGGCT GC-ACG--CG

350 s GCC-GCAC-- -G----AGAA A-GAG-CAAT AA---CAGGT CTG--TG-AT GCCCTTA--- -GA--TGGAC G----GGGCT GC-ACG--CG

358 s GCC-GCAC-- -G----AGAA A-GAG-CAAT AA---CAGGT CTG--TG-AT GCCCTTA--- -GA--TGGAC G----GGGCT GC-ACG--CG

367 s GCC-GCAC-- -G----AGAA A-GAG-CAAT AA---CAGGT CTG--TG-AT GCCCTTA--- -GA--TGGAC G----GGGCT GC-ACG--CG

375 d GCC-GCAC-- -G----AGAA A-GAG-CAAT AA---CAGGT CTG--TG-AT GCCCTTA--- -GA--TGGAC G----GGGCT GC-ACG--CG

375 s GCC-GCAC-- -G----AGAA A-GAG-CAAT AA---CAGGT CTG--TG-AT GCCCTTA--- -GA--TGGAC G----GGGCT GC-ACG--CG

375 si GCC-GCAC-- -G----AGAA A-GAG-CAAT AA---CAGGT CTG--TG-AT GCCCTTA--- -GA--TGGAC G----GGGCT GC-ACG--CG

454 s GCC-GCAC-- -G----AGAA A-GAG-CAAT AA---CAGGT CTG--TG-AT GCCCTTA--- -GA--TGGAC G----GGGCT GC-ACG--CG

516 s GCC-GCAC-- -G----AGAA A-GAG-CAAT AA---CAGGT CTG--TG-AT GCCCTTA--- -GA--TGGAC G----GGGCT GC-ACG--CG

561 s GCC-GCAC-- -G----AGAA A-GAG-CAAT AA---CAGGT CTG--TG-AT GCCCTTA--- -GA--TGGAC G----GGGCT GC-ACG--CG

615 d GCC-GCAC-- -G----AGAA A-GAG-CAAT AA---CAGGT CTG--TG-AT GCCCTTA--- -GA--TGGAC G----GGGCT GC-ACG--CG

615 s GCC-GCAC-- -G----AGAA A-GAG-CAAT AA---CAGGT CTG--TG-AT GCCCTTA--- -GA--TGGAC G----GGGCT GC-ACG--CG

64 s GCC-GCAC-- -G----AGAA A-GAG-CAAT AA---CAGGT CTG--TG-AT GCCCTTA--- -GA--TGGAC G----GGGCT GC-ACG--CG

658 d GCC-GCAC-- -G----AGAA A-GAG-CAAT AA---CAGGT CTG--TG-AT GCCCTTA--- -GA--TGGAC G----GGGCT GC-ACG--CG

658 s GCC-GCAC-- -G----AGAA A-GAG-CAAT AA---CAGGT CTG--TG-AT GCCCTTA--- -GA--TGGAC G----GGGCT GC-ACG--CG

662 d GCC-GCAC-- -G----AGAA A-GAG-CAAT AA---CAGGT CTG--TG-AT GCCCTTA--- -GA--TGGAC G----GGGCT GC-ACG--CG

662 s GCC-GCAC-- -G----AGAA A-GAG-CAAT AA---CAGGT CTG--TG-AT GCCCTTA--- -GA--TGGAC G----GGGCT GC-ACG--CG

757 s GCC-GCAC-- -G----AGAA A-GAG-CAAT AA---CAGGT CTG--TG-AT GCCCTTA--- -GA--TGGAC G----GGGCT GC-ACG--CG

768 d GCC-GCAC-- -G----AGAA A-GAG-CAAT AA---CAGGT CTG--TG-AT GCCCTTA--- -GA--TGGAC G----GGGCT GC-ACG--CG

768 s GCC-GCAC-- -G----AGAA A-GAG-CAAT AA---CAGGT CTG--TG-AT GCCCTTA--- -GA--TGGAC G----GGGCT GC-ACG--CG

796 d GCC-GCAC-- -G----AGAA A-GAG-CAAT AA---CAGGT CTG--TG-AT GCCCTTA--- -GA--TGGAC G----GGGCT GC-ACG--CG

796 s GCC-GCAC-- -G----AGAA A-GAG-CAAT AA---CAGGT CTG--TG-AT GCCCTTA--- -GA--TGGAC G----GGGCT GC-ACG--CG

798 d GCC-GCAC-- -G----AGAA A-GAG-CAAT AA---CAGGT CTG--TG-AT GCCCTTA--- -GA--TGGAC G----GGGCT GC-ACG--CG

798 s GCC-GCAC-- -G----AGAA A-GAG-CAAT AA---CAGGT CTG--TG-AT GCCCTTA--- -GA--TGGAC G----GGGCT GC-ACG--CG

8 si GCC-GCAC-- -G----AGAA A-GAG-CAAT AA---CAGGT CTG--TG-AT GCCCTTA--- -GA--TGGAC G----GGGCT GC-ACG--CG

805 s GCC-GCAC-- -G----AGAA A-GAG-CAAT AA---CAGGT CTG--TG-AT GCCCTTA--- -GA--TGGAC G----GGGCT GC-ACG--CG

811 s GCC-GCAC-- -G----AGAA A-GAG-CAAT AA---CAGGT CTG--TG-AT GCCCTTA--- -GA--TGGAC G----GGGCT GC-ACG--CG

92 s GCC-GCAC-- -G----AGAA A-GAG-CAAT AA---CAGGT CTG--TG-AT GCCCTTA--- -GA--TGGAC G----GGGCT GC-ACG--CG

94 s GCC-GCAC-- -G----AGAA A-GAG-CAAT AA---CAGGT CTG--TG-AT GCCCTTA--- -GA--TGGAC G----GGGCT GC-ACG--CG

230 si GCC-GCAC-- -G----AGAT T-GAG-CGAT AA---CAGGT CTG--TG-AT GCCCTTA--- -GA--TGTCC G----GGGCT GC-ACG--CG

454 d GCC-GCAC-- -G----AGAT T-GAG-CGAT AA---CAGGT CTG--TG-AT GCCCTTA--- -GA--TGTCC G----GGGCT GC-ACG--CG

332 d GCC-GCAC-- -G----AGAT T-GAG-CGAT AA---CAGGT CTG--TG-AT GCCCTTA--- -GA--TGTCC G----GGGCT GC-ACG--CG

454 s GCC-GCAC-- -G----AGAT T-GAG-CGAT AA---CAGGT CTG--TG-AT GCCCTTA--- -GA--TGTCC G----GGGCT GC-ACG--CG

621 d GCC-GCAC-- -G----AGAT T-GAG-CGAT AA---CAGGT CTG--TG-AT GCCCTTA--- -GA--TGTCC G----GGGCT GC-ACG--CG

64 si GCC-GCAC-- -G----AGAT T-GAG-CGAT AA---CAGGT CTG--TG-AT GCCCTTA--- -GA--TGTCC G----GGGCT GC-ACG--CG

621 d male M. patens GCC-GCAC-- -G----AGAT T-GAG-CGAT AA---CAGGT CTG--TG-AT GCCCTTA--- -GA--TGTCC G----GGGCT GC-ACG--CG

LC052360.1 A. putorii GCC-GCAC-- -G----AGAA A-GAG-CAAT AA---CAGGT CTG--TG-AT GCCCTTA--- -GA--TGGAC G----GGGCT GC-ACG--CG

JX456630.1 E. boehmi GCC-GCAC-- -G----AGAA A-GAG-CAAT AA---CAGGT CTG--TG-AT GCCCTTA--- -GA--TGGGC G----GGGCT GC-ACG--CG

KX962352.1 C. plica GCC-GCAC-- -G----AGAA A-GAG-CAAT AA---CAGGT CTG--TG-AT GCCCTTA--- -GA--TGGAC G----GGGCT GC-ACG--CG

MF287972.1 C. hepaticum GCC-GCAC-- -G----AGAA A-GAG-CAAT AA---CAGGT CTG--TG-AT GCCCTTA--- -GA--TGGAC G----GGGCT GC-ACG--CG

KC753538.1 C. splenaecum GCC-GCAC-- -G----AGAA A-GAG-CAAT AA---CAGGT CTG--TG-AT GCCCTTA--- -GA--TGGAC G----GGGCT GC-ACG--CG

KC341985.1 T. vulpis GCC-GCAC-- -G----AGAA A-GAG-CAAT AA---CAGGT CTG--TG-AT GCCCTTA--- -GA--TGTAC G----GGGCT GC-ACG--CG

550 560 570 580 590 600 610 620 630

....|....| ....|....| ....|....| ....|....| ....|....| ....|....| ....|....| ....|....| ....|....|

131 s TG-CTACA-- CTGATGGCA- CCAA--CATG CGTT-CAAGC CTGGCCT--- -GAAGAG--- ----GTCGGG T-----AATC GAAT--GAAA

19 s TG-CTACA-- CTGATGGCA- CCAA--CATG CGTT-CAAGC CTGGCCT--- -GAAGAG--- ----GTCGGG T-----AATC GAAT--GAAA

19 si TG-CTACA-- CTGATGGCA- CCAA--CATG CGTT-CAAGC CTGGCCT--- -GAAGAG--- ----GTCGGG T-----AATC GAAT--GAAA

21 s TG-CTACA-- CTGATGGCA- CCAA--CATG CGTT-CAAGC CTGGCCT--- -GAAGAG--- ----GTCGGG T-----AATC GAAT--GAAA

230 s TG-CTACA-- CTGATGGCA- CCAA--CATG CGTT-CAAGC CTGGCCT--- -GAAGAG--- ----GTCGGG T-----AATC GAAT--GAAA

236 s TG-CTACA-- CTGATGGCA- CCAA--CATG CGTT-CAAGC CTGGCCT--- -GAAGAG--- ----GTCGGG T-----AATC GAAT--GAAA

238 s TG-CTACA-- CTGATGGCA- CCAA--CATG CGTT-CAAGC CTGGCCT--- -GAAGAG--- ----GTCGGG T-----AATC GAAT--GAAA

246 s TG-CTACA-- CTGATGGCA- CCAA--CATG CGTT-CAAGC CTGGCCT--- -GAAGAG--- ----GTCGGG T-----AATC GAAT--GAAA

248 d TG-CTACA-- CTGATGGCA- CCAA--CATG CGTT-CAAGC CTGGCCT--- -GAAGAG--- ----GTCGGG T-----AATC GAAT--GAAA

248 s TG-CTACA-- CTGATGGCA- CCAA--CATG CGTT-CAAGC CTGGCCT--- -GAAGAG--- ----GTCGGG T-----AATC GAAT--GAAA

249 s TG-CTACA-- CTGATGGCA- CCAA--CATG CGTT-CAAGC CTGGCCT--- -GAAGAG--- ----GTCGGG T-----AATC GAAT--GAAA

255 s TG-CTACA-- CTGATGGCA- CCAA--CATG CGTT-CAAGC CTGGCCT--- -GAAGAG--- ----GTCGGG T-----AATC GAAT--GAAA

258 s TG-CTACA-- CTGATGGCA- CCAA--CATG CGTT-CAAGC CTGGCCT--- -GAAGAG--- ----GTCGGG T-----AATC GAAT--GAAA

27 s TG-CTACA-- CTGATGGCA- CCAA--CATG CGTT-CAAGC CTGGCCT--- -GAAGAG--- ----GTCGGG T-----AATC GAAT--GAAA

28 s TG-CTACA-- CTGATGGCA- CCAA--CATG CGTT-CAAGC CTGGCCT--- -GAAGAG--- ----GTCGGG T-----AATC GAAT--GAAA

290 d TG-CTACA-- CTGATGGCA- CCAA--CATG CGTT-CAAGC CTGGCCT--- -GAAGAG--- ----GTCGGG T-----AATC GAAT--GAAA

290 s TG-CTACA-- CTGATGGCA- CCAA--CATG CGTT-CAAGC CTGGCCT--- -GAAGAG--- ----GTCGGG T-----AATC GAAT--GAAA

294 s TG-CTACA-- CTGATGGCA- CCAA--CATG CGTT-CAAGC CTGGCCT--- -GAAGAG--- ----GTCGGG T-----AATC GAAT--GAAA

297 s TG-CTACA-- CTGATGGCA- CCAA--CATG CGTT-CAAGC CTGGCCT--- -GAAGAG--- ----GTCGGG T-----AATC GAAT--GAAA

300 s TG-CTACA-- CTGATGGCA- CCAA--CATG CGTT-CAAGC CTGGCCT--- -GAAGAG--- ----GTCGGG T-----AATC GAAT--GAAA

332 s TG-CTACA-- CTGATGGCA- CCAA--CATG CGTT-CAAGC CTGGCCT--- -GAAGAG--- ----GTCGGG T-----AATC GAAT--GAAA

342 s TG-CTACA-- CTGATGGCA- CCAA--CATG CGTT-CAAGC CTGGCCT--- -GAAGAG--- ----GTCGGG T-----AATC GAAT--GAAA

350 d TG-CTACA-- CTGATGGCA- CCAA--CATG CGTT-CAAGC CTGGCCT--- -GAAGAG--- ----GTCGGG T-----AATC GAAT--GAAA

350 s TG-CTACA-- CTGATGGCA- CCAA--CATG CGTT-CAAGC CTGGCCT--- -GAAGAG--- ----GTCGGG T-----AATC GAAT--GAAA

358 s TG-CTACA-- CTGATGGCA- CCAA--CATG CGTT-CAAGC CTGGCCT--- -GAAGAG--- ----GTCGGG T-----AATC GAAT--GAAA

367 s TG-CTACA-- CTGATGGCA- CCAA--CATG CGTT-CAAGC CTGGCCT--- -GAAGAG--- ----GTCGGG T-----AATC GAAT--GAAA

375 d TG-CTACA-- CTGATGGCA- CCAA--CATG CGTT-CAAGC CTGGCCT--- -GAAGAG--- ----GTCGGG T-----AATC GAAT--GAAA

375 s TG-CTACA-- CTGATGGCA- CCAA--CATG CGTT-CAAGC CTGGCCT--- -GAAGAG--- ----GTCGGG T-----AATC GAAT--GAAA

375 si TG-CTACA-- CTGATGGCA- CCAA--CATG CGTT-CAAGC CTGGCCT--- -GAAGAG--- ----GTCGGG T-----AATC GAAT--GAAA

454 s TG-CTACA-- CTGATGGCA- CCAA--CATG CGTT-CAAGC CTGGCCT--- -GAAGAG--- ----GTCGGG T-----AATC GAAT--GAAA

516 s TG-CTACA-- CTGATGGCA- CCAA--CATG CGTT-CAAGC CTGGCCT--- -GAAGAG--- ----GTCGGG T-----AATC GAAT--GAAA

561 s TG-CTACA-- CTGATGGCA- CCAA--CATG CGTT-CAAGC CTGGCCT--- -GAAGAG--- ----GTCGGG T-----AATC GAAT--GAAA

615 d TG-CTACA-- CTGATGGCA- CCAA--CATG CGTT-CAAGC CTGGCCT--- -GAAGAG--- ----GTCGGG T-----AATC GAAT--GAAA

615 s TG-CTACA-- CTGATGGCA- CCAA--CATG CGTT-CAAGC CTGGCCT--- -GAAGAG--- ----GTCGGG T-----AATC GAAT--GAAA

64 s TG-CTACA-- CTGATGGCA- CCAA--CATG CGTT-CAAGC CTGGCCT--- -GAAGAG--- ----GTCGGG T-----AATC GAAT--GAAA

658 d TG-CTACA-- CTGATGGCA- CCAA--CATG CGTT-CAAGC CTGGCCT--- -GAAGAG--- ----GTCGGG T-----AATC GAAT--GAAA

658 s TG-CTACA-- CTGATGGCA- CCAA--CATG CGTT-CAAGC CTGGCCT--- -GAAGAG--- ----GTCGGG T-----AATC GAAT--GAAA

662 d TG-CTACA-- CTGATGGCA- CCAA--CATG CGTT-CAAGC CTGGCCT--- -GAAGAG--- ----GTCGGG T-----AATC GAAT--GAAA

662 s TG-CTACA-- CTGATGGCA- CCAA--CATG CGTT-CAAGC CTGGCCT--- -GAAGAG--- ----GTCGGG T-----AATC GAAT--GAAA

757 s TG-CTACA-- CTGATGGCA- CCAA--CATG CGTT-CAAGC CTGGCCT--- -GAAGAG--- ----GTCGGG T-----AATC GAAT--GAAA

768 d TG-CTACA-- CTGATGGCA- CCAA--CATG CGTT-CAAGC CTGGCCT--- -GAAGAG--- ----GTCGGG T-----AATC GAAT--GAAA

768 s TG-CTACA-- CTGATGGCA- CCAA--CATG CGTT-CAAGC CTGGCCT--- -GAAGAG--- ----GTCGGG T-----AATC GAAT--GAAA

796 d TG-CTACA-- CTGATGGCA- CCAA--CATG CGTT-CAAGC CTGGCCT--- -GAAGAG--- ----GTCGGG T-----AATC GAAT--GAAA

796 s TG-CTACA-- CTGATGGCA- CCAA--CATG CGTT-CAAGC CTGGCCT--- -GAAGAG--- ----GTCGGG T-----AATC GAAT--GAAA

798 d TG-CTACA-- CTGATGGCA- CCAA--CATG CGTT-CAAGC CTGGCCT--- -GAAGAG--- ----GTCGGG T-----AATC GAAT--GAAA

798 s TG-CTACA-- CTGATGGCA- CCAA--CATG CGTT-CAAGC CTGGCCT--- -GAAGAG--- ----GTCGGG T-----AATC GAAT--GAAA

8 si TG-CTACA-- CTGATGGCA- CCAA--CATG CGTT-CAAGC CTGGCCT--- -GAAGAG--- ----GTCGGG T-----AATC GAAT--GAAA

805 s TG-CTACA-- CTGATGGCA- CCAA--CATG CGTT-CAAGC CTGGCCT--- -GAAGAG--- ----GTCGGG T-----AATC GAAT--GAAA

811 s TG-CTACA-- CTGATGGCA- CCAA--CATG CGTT-CAAGC CTGGCCT--- -GAAGAG--- ----GTCGGG T-----AATC GAAT--GAAA

92 s TG-CTACA-- CTGATGGCA- CCAA--CATG CGTT-CAAGC CTGGCCT--- -GAAGAG--- ----GTCGGG T-----AATC GAAT--GAAA

94 s TG-CTACA-- CTGATGGCA- CCAA--CATG CGTT-CAAGC CTGGCCT--- -GAAGAG--- ----GTCGGG T-----AATC GAAT--GAAA

230 si CG-CTACA-- ATGGAAG-AA TCAG--CTGG CCTA--T-CC ATTGCC---- GAAAGGC--- ----ATTGG- TA----AACC G--TTGAAAC

454 d CG-CTACA-- ATGGAAG-AA TCAG--CTGG CCTA--T-CC ATTGCC---- GAAAGGC--- ----ATTGG- TA----AACC G--TTGAAAC

332 d CG-CTACA-- ATGGAAG-AA TCAG--CTGG CCTA--T-CC ATTGCC---- GAAAGGC--- ----ATTGG- TA----AACC G--TTGAAAC

454 s CG-CTACA-- ATGGAAG-AA TCAG--CTGG CCTA--T-CC ATTGCC---- GAAAGGC--- ----ATTGG- TA----AACC G--TTGAAAC

621 d CG-CTACA-- ATGGAAG-AA TCAG--CTGG CCTA--T-CC ATTGCC---- GAAAGGC--- ----ATTGG- TA----AACC G--TTGAAAC

64 si CG-CTACA-- ATGGAAG-AA TCAG--CTGG CCTA--T-CC ATTGCC---- GAAAGGT--- ----ATTGG- TA----AACC G--TTGAAAC

621 d male M. patens CG-CTACA-- ATGGAAG-AA TCAG--CTGG CCTA--T-CC ATTGCC---- GAAAGGC--- ----ATTGG- TA----AACC G--TTGAAAC

LC052360.1 A. putorii TG-CTACA-- CTGATGGCA- CCAA--CATG CGTT-CAAGC CTGGCCT--- -GAAGAG--- ----GTCGGG T-----AATC GAAT--GAAA

JX456630.1 E. boehmi TG-CTACA-- CTGATGGCA- GCAA--CGTG CGTC-CAAGC CTGACCT--- -GTGAAG--- ----GTCAGG A-----AATC GATT--GAAA

KX962352.1 C. plica TG-CTACA-- CTGATGGCA- CCAA--CGTG CGTT-CAAGC CTGGCTT--- -GAAGAG--- ----GTCAGG T-----AATC GAAT--GAAA

MF287972.1 C. hepaticum TG-CTACA-- CTGACGGCA- CCAG--CGTG CGTT-CAAGC CTGTCTT--- -GAAGAG--- ----GTCAGG T-----AATC GATT--GAAA

KC753538.1 C. splenaecum TG-CTACA-- CTGATGGCA- TCAA--CGTG CGTT-CAAGC CTGGCCT--- -GAAGAG--- ----GTCAGG A-----AATC GAAT--GAAA

KC341985.1 T. vulpis TG-CTACA-- CTGACGGCG- TCAG--CGTG CGTT-CAAGC CCGGCCT--- -GGCAAG--- ----GTCGGG A-----AATC GGTT--GAAA

640 650 660 670 680 690 700 710 720

....|....| ....|....| ....|....| ....|....| ....|....| ....|....| ....|....| ....|....| ....|....|

131 s TGTCCG---T CGT-GACTGG GAC----AGG GAA-TTGCAA ----TTA-TT TC----CCTC ---GAACG-A GGAA--TTCC C--AGTAAG-

19 s TGTCCG---T CGT-GACTGG GAC----AGG GAA-TTGCAA ----TTA-TT TC----CCTC ---GAACG-A GGAA--TTCC C--AGTAAG-

19 si TGTCCG---T CGT-GACTGG GAC----AGG GAA-TTGCAA ----TTA-TT TC----CCTC ---GAACG-A GGAA--TTCC C--AGTAAG-

21 s TGTCCG---T CGT-GACTGG GAC----AGG GAA-TTGCAA ----TTA-TT TC----CCTC ---GAACG-A GGAA--TTCC C--AGTAAG-

230 s TGTCCG---T CGT-GACTGG GAC----AGG GAA-TTGCAA ----TTA-TT TC----CCTC ---GAACG-A GGAA--TTCC C--AGTAAG-

236 s TGTCCG---T CGT-GACTGG GAC----AGG GAA-TTGCAA ----TTA-TT TC----CCTC ---GAACG-A GGAA--TTCC C--AGTAAG-

238 s TGTCCG---T CGT-GACTGG GAC----AGG GAA-TTGCAA ----TTA-TT TC----CCTC ---GAACG-A GGAA--TTCC C--AGTAAG-

246 s TGTCCG---T CGT-GACTGG GAC----AGG GAA-TTGCAA ----TTA-TT TC----CCTC ---GAACG-A GGAA--TTCC C--AGTAAG-

248 d TGTCCG---T CGT-GACTGG GAC----AGG GAA-TTGCAA ----TTA-TT TC----CCTC ---GAACG-A GGAA--TTCC C--AGTAAG-

248 s TGTCCG---T CGT-GACTGG GAC----AGG GAA-TTGCAA ----TTA-TT TC----CCTC ---GAACG-A GGAA--TTCC C--AGTAAG-

249 s TGTCCG---T CGT-GACTGG GAC----AGG GAA-TTGCAA ----TTA-TT TC----CCTC ---GAACG-A GGAA--TTCC C--AGTAAG-

255 s TGTCCG---T CGT-GACTGG GAC----AGG GAA-TTGCAA ----TTA-TT TC----CCTC ---GAACG-A GGAA--TTCC C--AGTAAG-

258 s TGTCCG---T CGT-GACTGG GAC----AGG GAA-TTGCAA ----TTA-TT TC----CCTC ---GAACG-A GGAA--TTCC C--AGTAAG-

27 s TGTCCG---T CGT-GACTGG GAC----AGG GAA-TTGCAA ----TTA-TT TC----CCTC ---GAACG-A GGAA--TTCC C--AGTAAA-

28 s TGTCCG---T CGT-GACTGG GAC----AGG GAA-TTGCAA ----TTA-TT TC----CCTC ---GAACG-A GGAA--TTCC C--AGTAAG-

290 d TGTCCG---T CGT-GACTGG GAC----AGG GAA-TTGCAA ----TTA-TT TC----CCTC ---GAACG-A GGAA--TTCC C--AGTAAG-

290 s TGTCCG---T CGT-GACTGG GAC----AGG GAA-TTGCAA ----TTA-TT TC----CCTC ---GAACG-A GGAA--TTCC C--AGTAAG-

294 s TGTCCG---T CGT-GACTGG GAC----AGG GAA-TTGCAA ----TTA-TT TC----CCTC ---GAACG-A GGAA--TTCC C--AGTAAG-

297 s TGTCCG---T CGT-GACTGG GAC----AGG GAA-TTGCAA ----TTA-TT TC----CCTC ---GAACG-A GGAA--TTCC C--AGTAAG-

300 s TGTCCG---T CGT-GACTGG GAC----AGG GAA-TTGCAA ----TTA-TT TC----CCTC ---GAACG-A GGAA--TTCC C--AGTAAG-

332 s TGTCCG---T CGT-GACTGG GAC----AGG GAA-TTGCAA ----TTA-TT TC----CCTC ---GAACG-A GGAA--TTCC C--AGTAAG-

342 s TGTCCG---T CGT-GACTGG GAC----AGG GAA-TTGCAA ----TTA-TT TC----CCTC ---GAACG-A GGAA--TTCC C--AGTAAG-

350 d TGTCCG---T CGT-GACTGG GAC----AGG GAA-TTGCAA ----TTA-TT TC----CCTC ---GAACG-A GGAA--TTCC C--AGTAAG-

350 s TGTCCG---T CGT-GACTGG GAC----AGG GAA-TTGCAA ----TTA-TT TC----CCTC ---GAACG-A GGAA--TTCC C--AGTAAG-

358 s TGTCCG---T CGT-GACTGG GAC----AGG GAA-TTGCAA ----TTA-TT TC----CCTC ---GAACG-A GGAA--TTCC C--AGTAAG-

367 s TGTCCG---T CGT-GACTGG GAC----AGG GAA-TTGCAA ----TTA-TT TC----CCTC ---GAACG-A GGAA--TTCC C--AGTAAG-

375 d TGTCCG---T CGT-GACTGG GAC----AGG GAA-TTGCAA ----TTA-TT TC----CCTC ---GAACG-A GGAA--TTCC C--AGTAAG-

375 s TGTCCG---T CGT-GACTGG GAC----AGG GAA-TTGCAA ----TTA-TT TC----CCTC ---GAACG-A GGAA--TTCC C--AGTAAG-

375 si TGTCCG---T CGT-GACTGG GAC----AGG GAA-TTGCAA ----TTA-TT TC----CCTC ---GAACG-A GGAA--TTCC C--AGTAAG-

454 s TGTCCG---T CGT-GACTGG GAC----AGG GAA-TTGCAA ----TTA-TT TC----CCTC ---GAACG-A GGAA--TTCC C--AGTAAG-

516 s TGTCCG---T CGT-GACTGG GAC----AGG GAA-TTGCAA ----TTA-TT TC----CCTC ---GAACG-A GGAA--TTCC C--AGTAAG-

561 s TGTCCG---T CGT-GACTGG GAC----AGG GAA-TTGCAA ----TTA-TT TC----CCTC ---GAACG-A GGAA--TTCC C--AGTAAG-

615 d TGTCCG---T CGT-GACTGG GAC----AGG GAA-TTGCAA ----TTA-TT TC----CCTC ---GAACG-A GGAA--TTCC C--AGTAAG-

615 s TGTCCG---T CGT-GACTGG GAC----AGG GAA-TTGCAA ----TTA-TT TC----CCTC ---GAACG-A GGAA--TTCC C--AGTAAG-

64 s TGTCCG---T CGT-GACTGG GAC----AGG GAA-TTGCAA ----TTA-TT TC----CCTC ---GAACG-A GGAA--TTCC C--AGTAAG-

658 d TGTCCG---T CGT-GACTGG GAC----AGG GAA-TTGCAA ----TTA-TT TC----CCTC ---GAACG-A GGAA--TTCC C--AGTAAG-

658 s TGTCCG---T CGT-GACTGG GAC----AGG GAA-TTGCAA ----TTA-TT TC----CCTC ---GAACG-A GGAA--TTCC C--AGTAAG-

662 d TGTCCG---T CGT-GACTGG GAC----AGG GAA-TTGCAA ----TTA-TT TC----CCTC ---GAACG-A GGAA--TTCC C--AGTAAG-

662 s TGTCCG---T CGT-GACTGG GAC----AGG GAA-TTGCAA ----TTA-TT TC----CCTC ---GAACG-A GGAA--TTCC C--AGTAAG-

757 s TGTCCG---T CGT-GACTGG GAC----AGG GAA-TTGCAA ----TTA-TT TC----CCTC ---GAACG-A GGAA--TTCC C--AGTAAG-

768 d TGTCCG---T CGT-GACTGG GAC----AGG GAA-TTGCAA ----TTA-TT TC----CCTC ---GAACG-A GGAA--TTCC C--AGTAAG-

768 s TGTCCG---T CGT-GACTGG GAC----AGG GAA-TTGCAA ----TTA-TT TC----CCTC ---GAACG-A GGAA--TTCC C--AGTAAG-

796 d TGTCCG---T CGT-GACTGG GAC----AGG GAA-TTGCAA ----TTA-TT TC----CCTC ---GAACG-A GGAA--TTCC C--AGTAAG-

796 s TGTCCG---T CGT-GACTGG GAC----AGG GAA-TTGCAA ----TTA-TT TC----CCTC ---GAACG-A GGAA--TTCC C--AGTAAG-

798 d TGTCCG---T CGT-GACTGG GAC----AGG GAA-TTGCAA ----TTA-TT TC----CCTC ---GAACG-A GGAA--TTCC C--AGTAAG-

798 s TGTCCG---T CGT-GACTGG GAC----AGG GAA-TTGCAA ----TTA-TT TC----CCTC ---GAACG-A GGAA--TTCC C--AGTAAG-

8 si TGTCCG---T CGT-GACTGG GAC----AGG GAA-TTGCAA ----TTA-TT TC----CCTC ---GAACG-A GGAA--TTCC C--AGTAAG-

805 s TGTCCG---T CGT-GACTGG GAC----AGG GAA-TTGCAA ----TTA-TT TC----CCTC ---GAACG-A GGAA--TTCC C--AGTAAG-

811 s TGTCCG---T CGT-GACTGG GAC----AGG GAA-TTGCAA ----TTA-TT TC----CCTC ---GAACG-A GGAA--TTCC C--AGTAAG-

92 s TGTCCG---T CGT-GACTGG GAC----AGG GAA-TTGCAA ----TTA-TT TC----CCTC ---GAACG-A GGAA--TTCC C--AGTAAG-

94 s TGTCCG---T CGT-GACTGG GAC----AGG GAA-TTGCAA ----TTA-TT TC----CCTC ---GAACG-A GGAA--TTCC C--AGTAAG-

230 si TCTTCC---- -GTG-ACCGG GATA----GG GAA-TTGTAA ----TTA-TT TC----CCTT ---GAACGAG G-AA--TTCC T--AGTAAG-

454 d TCTTCC---- -GTG-ACCGG GATA----GG GAA-TTGTAA ----TTA-TT TC----CCTT ---GAACGAG G-AA--TTCC T--AGTAAG-

332 d TCTTCC---- -GTG-ACCGG GATA----GG GAA-TTGTAA ----TTA-TT TC----CCTT ---GAACGAG G-AA--TTCC T--AGTAAG-

454 s TCTTCC---- -GTG-ACCGG GATA----GG GAA-TTGTAA ----TTA-TT TC----CCTT ---GAACGAG G-AA--TTCC T--AGTAAG-

621 d TCTTCCC--- -GTG-ACCGG GATA----GG GAA-TTGTAA ----TTA-TT TC----CCTT ---GAACGAG G-AA--TTCC TT-AGTAAG-

64 si TCTTCC---- -GTG-ACCGG GATA----GG GAA-TTGTAA ----TTA-TT TC----CCTT ---GAACGAG G-AA--TTCC T--AGTAAG-

621 d male M. patens TCTTCC---- -GTG-ACCGG GATA----GG GAA-TTGTAA ----TTA-TT TC----CCTT ---GAACGAG G-AA--TTCC T--AGTAAG-

LC052360.1 A. putorii TGTCCG---T CGT-GACTGG GAC----AGG GAA-TTGCAA ----TTA-TT TC----CCTC ---GAACG-A GGAA--TTCC C--AGTAAG-

JX456630.1 E. boehmi TGTCCG---T CGT-GACTGG GAT----AGG GAA-TTGCAA ----TTA-TT TC----CCTT ---GAACG-C GGAA--TTCC C--AGTAAG-

KX962352.1 C. plica TGTCCG---T CGT-GACTGG GAC----AGG GAA-TTGCAA ----TTA-TT TC----CCTC ---GAACG-A GGAA--TTCC C--AGTAAG-

MF287972.1 C. hepaticum TGTCCG---T CGT-GACTGG GAC----AGG GAA-TTGCAA ----TTA-TT TC----CCTC ---GAACG-A GGAA--TTCC C--AGTAAG-

KC753538.1 C. splenaecum TGTCCG---T CGT-GACTGG GAC----AGG GAA-TTGCAA ----TTA-TT TC----CCTC ---GAACG-A GGAA--TTCC C--AGTAAG-

KC341985.1 T. vulpis CGTTC----T CGT-GACTGG GAC----AGG GAA-TTGCAA ----TTA-TT TC----CCTC ---GAACG-A GGAA--TTCC C--AGTAAG-

730 740 750 760 770 780 790 800 810

....|....| ....|....| ....|....| ....|....| ....|....| ....|....| ....|....| ....|....| ....|....|

131 s ---CGCG--- AGTCAT---- CAGCTT-G-C GTTG------ ---------- ---------- ---------- ---------- ----------

19 s ---CGCG--- AGTCAT---- CAGCTT-G-C GTTG------ ---------- ---------- ---------- ---------- ----------

19 si ---CGCG--- AGTCAT---- CAGCTT-G-C GTTG------ ---------- ---------- ---------- ---------- ----------

21 s ---CGCG--- AGTCAT---- CAGCTT-G-C GTTG------ ---------- ---------- ---------- ---------- ----------

230 s ---CGCG--- AGTCAT---- CAGCTT-G-C GTTG------ ---------- ---------- ---------- ---------- ----------

236 s ---CGCG--- AGTCAT---- CAGCTT-G-C GTTG------ ---------- ---------- ---------- ---------- ----------

238 s ---CGCG--- AGTCAT---- CAGCTT-G-C GTTG------ ---------- ---------- ---------- ---------- ----------

246 s ---CGCG--- AGTCAT---- CAGCTT-G-C GTTG------ ---------- ---------- ---------- ---------- ----------

248 d ---CGCG--- AGTCAT---- CAGCTT-G-C GTTG------ ---------- ---------- ---------- ---------- ----------

248 s ---CGCG--- AGTCAT---- CAGCTT-G-C GTTG------ ---------- ---------- ---------- ---------- ----------

249 s ---CGCG--- AGTCAT---- CAGCTT-G-C GTTG------ ---------- ---------- ---------- ---------- ----------

255 s ---CGCG--- AGTCAT---- CAGCTT-G-C GTTG------ ---------- ---------- ---------- ---------- ----------

258 s ---CGCG--- AGTCAT---- CAGCTT-G-C GTTG------ ---------- ---------- ---------- ---------- ----------

27 s ---CGCG--- AGTCAT---- CAGCTT-G-C GTTG------ ---------- ---------- ---------- ---------- ----------

28 s ---CGCG--- AGTCAT---- CAGCTT-G-C GTTG------ ---------- ---------- ---------- ---------- ----------

290 d ---CGCG--- AGTCAT---- CAGCTT-G-C GTTG------ ---------- ---------- ---------- ---------- ----------

290 s ---CGCG--- AGTCAT---- CAGCTT-G-C GTTG------ ---------- ---------- ---------- ---------- ----------

294 s ---CGCG--- AGTCAT---- CAGCTT-G-C GTTG------ ---------- ---------- ---------- ---------- ----------

297 s ---CGCG--- AGTCAT---- CAGCTT-G-C GTTG------ ---------- ---------- ---------- ---------- ----------

300 s ---CGCG--- AGTCAT---- CAGCTT-G-C GTTG------ ---------- ---------- ---------- ---------- ----------

332 s ---CGCG--- AGTCAT---- CAGCTT-G-C GTTG------ ---------- ---------- ---------- ---------- ----------

342 s ---CGCG--- AGTCAT---- CAGCTT-G-C GTTG------ ---------- ---------- ---------- ---------- ----------

350 d ---CGCG--- AGTCAT---- CAGCTT-G-C GTTG------ ---------- ---------- ---------- ---------- ----------

350 s ---CGCG--- AGTCAT---- CAGCTT-G-C GTTG------ ---------- ---------- ---------- ---------- ----------

358 s ---CGCG--- AGTCAT---- CAGCTT-G-C GTTG------ ---------- ---------- ---------- ---------- ----------

367 s ---CGCG--- AGTCAT---- CAGCTT-G-C GTTG------ ---------- ---------- ---------- ---------- ----------

375 d ---CGCG--- AGTCAT---- CAGCTT-G-C GTTG------ ---------- ---------- ---------- ---------- ----------

375 s ---CGCG--- AGTCAT---- CAGCTT-G-C GTTG------ ---------- ---------- ---------- ---------- ----------

375 si ---CGCG--- AGTCAT---- CAGCTT-G-C GTTG------ ---------- ---------- ---------- ---------- ----------

454 s ---CGCG--- AGTCAT---- CAGCTT-G-C GTTG------ ---------- ---------- ---------- ---------- ----------

516 s ---CGCG--- AGTCAT---- CAGCTT-G-C GTTG------ ---------- ---------- ---------- ---------- ----------

561 s ---CGCG--- AGTCAT---- CAGCTT-G-C GTTG------ ---------- ---------- ---------- ---------- ----------

615 d ---CGCG--- AGTCAT---- CAGCTT-G-C GTTG------ ---------- ---------- ---------- ---------- ----------

615 s ---CGCG--- AGTCAT---- CAGCTT-G-C GTTG------ ---------- ---------- ---------- ---------- ----------

64 s ---CGCG--- AGTCAT---- CAGCTT-G-C GTTG------ ---------- ---------- ---------- ---------- ----------

658 d ---CGCG--- AGTCAT---- CAGCTT-G-C GTTG------ ---------- ---------- ---------- ---------- ----------

658 s ---CGCG--- AGTCAT---- CAGCTT-G-C GTTG------ ---------- ---------- ---------- ---------- ----------

662 d ---CGCG--- AGTCAT---- CAACTT-G-C GT-G------ ---------- ---------- ---------- ---------- ----------

662 s ---CGCG--- AGTCAT---- CAGCTT-G-C GTTG------ ---------- ---------- ---------- ---------- ----------

757 s ---CGCG--- AGTCAT---- CAGCTT-G-C GTTG------ ---------- ---------- ---------- ---------- ----------

768 d ---CGCG--- AGTCAT---- CAGCTT-G-C GTTG------ ---------- ---------- ---------- ---------- ----------

768 s ---CGCG--- AGTCAT---- CAGCTT-G-C GTTG------ ---------- ---------- ---------- ---------- ----------

796 d ---CGCG--- AGTCAT---- CAGCTT-G-C GTTG------ ---------- ---------- ---------- ---------- ----------

796 s ---CGCG--- AGTCAT---- CAGCTT-G-C GTTG------ ---------- ---------- ---------- ---------- ----------

798 d ---CGCG--- AGTCAT---- CAGCTT-G-C GTTG------ ---------- ---------- ---------- ---------- ----------

798 s ---CGCG--- AGTCAT---- CAGCTT-G-C GTTG------ ---------- ---------- ---------- ---------- ----------

8 si ---CGCG--- AGTCAT---- CAGCTT-G-C GTTG------ ---------- ---------- ---------- ---------- ----------

805 s ---CGCG--- AGTCAT---- CAGCTT-G-C GTTG------ ---------- ---------- ---------- ---------- ----------

811 s ---CGCG--- AGTCAT---- CAGCTT-G-C GTTG------ ---------- ---------- ---------- ---------- ----------

92 s ---CGCG--- AGTCAT---- CAGCTT-G-C GTTG------ ---------- ---------- ---------- ---------- ----------

94 s ---CGCG--- AGTCAT---- CAGCTT-G-C GTTG------ ---------- ---------- ---------- ---------- ----------

230 si ---TGTG--- AGTCA--T-- CAGCT--CAC GCTG------ ---------- ---------- ---------- ---------- ----------

454 d ---TGTG--- AGTCA--T-- CAGCT--CAC GCTG------ ---------- ---------- ---------- ---------- ----------

332 d ---TGTG--- AGTCA--T-- CAGCT--CAC GCTG------ ---------- ---------- ---------- ---------- ----------

454 s ---TGTG--- AGTCA--T-- CAGCT--CAC GCTG------ ---------- ---------- ---------- ---------- ----------

621 d ---TGGG--- AGTCA--T-- CAGCT--CAC GCTG------ ---------- ---------- ---------- ---------- ----------

64 si ---TGTG--- AGTCA--T-- CAGCT--CAC GCTG------ ---------- ---------- ---------- ---------- ----------

621 d male M. patens ---TGTG--- AGTCA--T-- CAGCT--CAC GCTG------ ---------- ---------- ---------- ---------- ----------

LC052360.1 A. putorii ---------- ---------- ---------- ---------- ---------- ---------- ---------- ---------- ----------

JX456630.1 E. boehmi ---------- ---------- ---------- ---------- ---------- ---------- ---------- ---------- ----------

KX962352.1 C. plica ---------- ---------- ---------- ---------- ---------- ---------- ---------- ---------- ----------

MF287972.1 C. hepaticum ---------- ---------- ---------- ---------- ---------- ---------- ---------- ---------- ----------

KC753538.1 C. splenaecum ---------- ---------- ---------- ---------- ---------- ---------- ---------- ---------- ----------

KC341985.1 T. vulpis ---------- ---------- ---------- ---------- ---------- ---------- ---------- ---------- ----------

820 830 840 850 860 870 880 890 900

....|....| ....|....| ....|....| ....|....| ....|....| ....|....| ....|....| ....|....| ....|....|

131 s ---------- ---------- ---------- ---------- ---------- ---------- ---------- ---------- ----------

19 s ---------- ---------- ---------- ---------- ---------- ---------- ---------- ---------- ----------

19 si ---------- ---------- ---------- ---------- ---------- ---------- ---------- ---------- ----------

21 s ---------- ---------- ---------- ---------- ---------- ---------- ---------- ---------- ----------

230 s ---------- ---------- ---------- ---------- ---------- ---------- ---------- ---------- ----------

236 s ---------- ---------- ---------- ---------- ---------- ---------- ---------- ---------- ----------

238 s ---------- ---------- ---------- ---------- ---------- ---------- ---------- ---------- ----------

246 s ---------- ---------- ---------- ---------- ---------- ---------- ---------- ---------- ----------

248 d ---------- ---------- ---------- ---------- ---------- ---------- ---------- ---------- ----------

248 s ---------- ---------- ---------- ---------- ---------- ---------- ---------- ---------- ----------

249 s ---------- ---------- ---------- ---------- ---------- ---------- ---------- ---------- ----------

255 s ---------- ---------- ---------- ---------- ---------- ---------- ---------- ---------- ----------

258 s ---------- ---------- ---------- ---------- ---------- ---------- ---------- ---------- ----------

27 s ---------- ---------- ---------- ---------- ---------- ---------- ---------- ---------- ----------

28 s ---------- ---------- ---------- ---------- ---------- ---------- ---------- ---------- ----------

290 d ---------- ---------- ---------- ---------- ---------- ---------- ---------- ---------- ----------

290 s ---------- ---------- ---------- ---------- ---------- ---------- ---------- ---------- ----------

294 s ---------- ---------- ---------- ---------- ---------- ---------- ---------- ---------- ----------

297 s ---------- ---------- ---------- ---------- ---------- ---------- ---------- ---------- ----------

300 s ---------- ---------- ---------- ---------- ---------- ---------- ---------- ---------- ----------

332 s ---------- ---------- ---------- ---------- ---------- ---------- ---------- ---------- ----------

342 s ---------- ---------- ---------- ---------- ---------- ---------- ---------- ---------- ----------

350 d ---------- ---------- ---------- ---------- ---------- ---------- ---------- ---------- ----------

350 s ---------- ---------- ---------- ---------- ---------- ---------- ---------- ---------- ----------

358 s ---------- ---------- ---------- ---------- ---------- ---------- ---------- ---------- ----------

367 s ---------- ---------- ---------- ---------- ---------- ---------- ---------- ---------- ----------

375 d ---------- ---------- ---------- ---------- ---------- ---------- ---------- ---------- ----------

375 s ---------- ---------- ---------- ---------- ---------- ---------- ---------- ---------- ----------

375 si ---------- ---------- ---------- ---------- ---------- ---------- ---------- ---------- ----------

454 s ---------- ---------- ---------- ---------- ---------- ---------- ---------- ---------- ----------

516 s ---------- ---------- ---------- ---------- ---------- ---------- ---------- ---------- ----------

561 s ---------- ---------- ---------- ---------- ---------- ---------- ---------- ---------- ----------

615 d ---------- ---------- ---------- ---------- ---------- ---------- ---------- ---------- ----------

615 s ---------- ---------- ---------- ---------- ---------- ---------- ---------- ---------- ----------

64 s ---------- ---------- ---------- ---------- ---------- ---------- ---------- ---------- ----------

658 d ---------- ---------- ---------- ---------- ---------- ---------- ---------- ---------- ----------

658 s ---------- ---------- ---------- ---------- ---------- ---------- ---------- ---------- ----------

662 d ---------- ---------- ---------- ---------- ---------- ---------- ---------- ---------- ----------

662 s ---------- ---------- ---------- ---------- ---------- ---------- ---------- ---------- ----------

757 s ---------- ---------- ---------- ---------- ---------- ---------- ---------- ---------- ----------

768 d ---------- ---------- ---------- ---------- ---------- ---------- ---------- ---------- ----------

768 s ---------- ---------- ---------- ---------- ---------- ---------- ---------- ---------- ----------

796 d ---------- ---------- ---------- ---------- ---------- ---------- ---------- ---------- ----------

796 s ---------- ---------- ---------- ---------- ---------- ---------- ---------- ---------- ----------

798 d ---------- ---------- ---------- ---------- ---------- ---------- ---------- ---------- ----------

798 s ---------- ---------- ---------- ---------- ---------- ---------- ---------- ---------- ----------

8 si ---------- ---------- ---------- ---------- ---------- ---------- ---------- ---------- ----------

805 s ---------- ---------- ---------- ---------- ---------- ---------- ---------- ---------- ----------

811 s ---------- ---------- ---------- ---------- ---------- ---------- ---------- ---------- ----------

92 s ---------- ---------- ---------- ---------- ---------- ---------- ---------- ---------- ----------

94 s ---------- ---------- ---------- ---------- ---------- ---------- ---------- ---------- ----------

230 si ---------- ---------- ---------- ---------- ---------- ---------- ---------- ---------- ----------

454 d ---------- ---------- ---------- ---------- ---------- ---------- ---------- ---------- ----------

332 d ---------- ---------- ---------- ---------- ---------- ---------- ---------- ---------- ----------

454 s ---------- ---------- ---------- ---------- ---------- ---------- ---------- ---------- ----------

621 d ---------- ---------- ---------- ---------- ---------- ---------- ---------- ---------- ----------

64 si ---------- ---------- ---------- ---------- ---------- ---------- ---------- ---------- ----------

621 d male M. patens ---------- ---------- ---------- ---------- ---------- ---------- ---------- ---------- ----------

LC052360.1 A. putorii ---------- ---------- ---------- ---------- ---------- ---------- ---------- ---------- ----------

JX456630.1 E. boehmi ---------- ---------- ---------- ---------- ---------- ---------- ---------- ---------- ----------

KX962352.1 C. plica ---------- ---------- ---------- ---------- ---------- ---------- ---------- ---------- ----------

MF287972.1 C. hepaticum ---------- ---------- ---------- ---------- ---------- ---------- ---------- ---------- ----------

KC753538.1 C. splenaecum ---------- ---------- ---------- ---------- ---------- ---------- ---------- ---------- ----------

KC341985.1 T. vulpis ---------- ---------- ---------- ---------- ---------- ---------- ---------- ---------- ----------

910 920 930 940 950 960 970 980 990

....|....| ....|....| ....|....| ....|....| ....|....| ....|....| ....|....| ....|....| ....|....|

131 s ---------- ---------- ---------- ---------- ---------- ---------- ---------- ---------- ----------

19 s ---------- ---------- ---------- ---------- ---------- ---------- ---------- ---------- ----------

19 si ---------- ---------- ---------- ---------- ---------- ---------- ---------- ---------- ----------

21 s ---------- ---------- ---------- ---------- ---------- ---------- ---------- ---------- ----------

230 s ---------- ---------- ---------- ---------- ---------- ---------- ---------- ---------- ----------

236 s ---------- ---------- ---------- ---------- ---------- ---------- ---------- ---------- ----------

238 s ---------- ---------- ---------- ---------- ---------- ---------- ---------- ---------- ----------

246 s ---------- ---------- ---------- ---------- ---------- ---------- ---------- ---------- ----------

248 d ---------- ---------- ---------- ---------- ---------- ---------- ---------- ---------- ----------

248 s ---------- ---------- ---------- ---------- ---------- ---------- ---------- ---------- ----------

249 s ---------- ---------- ---------- ---------- ---------- ---------- ---------- ---------- ----------

255 s ---------- ---------- ---------- ---------- ---------- ---------- ---------- ---------- ----------

258 s ---------- ---------- ---------- ---------- ---------- ---------- ---------- ---------- ----------

27 s ---------- ---------- ---------- ---------- ---------- ---------- ---------- ---------- ----------

28 s ---------- ---------- ---------- ---------- ---------- ---------- ---------- ---------- ----------

290 d ---------- ---------- ---------- ---------- ---------- ---------- ---------- ---------- ----------

290 s ---------- ---------- ---------- ---------- ---------- ---------- ---------- ---------- ----------

294 s ---------- ---------- ---------- ---------- ---------- ---------- ---------- ---------- ----------

297 s ---------- ---------- ---------- ---------- ---------- ---------- ---------- ---------- ----------

300 s ---------- ---------- ---------- ---------- ---------- ---------- ---------- ---------- ----------

332 s ---------- ---------- ---------- ---------- ---------- ---------- ---------- ---------- ----------

342 s ---------- ---------- ---------- ---------- ---------- ---------- ---------- ---------- ----------

350 d ---------- ---------- ---------- ---------- ---------- ---------- ---------- ---------- ----------

350 s ---------- ---------- ---------- ---------- ---------- ---------- ---------- ---------- ----------

358 s ---------- ---------- ---------- ---------- ---------- ---------- ---------- ---------- ----------

367 s ---------- ---------- ---------- ---------- ---------- ---------- ---------- ---------- ----------

375 d ---------- ---------- ---------- ---------- ---------- ---------- ---------- ---------- ----------

375 s ---------- ---------- ---------- ---------- ---------- ---------- ---------- ---------- ----------

375 si ---------- ---------- ---------- ---------- ---------- ---------- ---------- ---------- ----------

454 s ---------- ---------- ---------- ---------- ---------- ---------- ---------- ---------- ----------

516 s ---------- ---------- ---------- ---------- ---------- ---------- ---------- ---------- ----------

561 s ---------- ---------- ---------- ---------- ---------- ---------- ---------- ---------- ----------

615 d ---------- ---------- ---------- ---------- ---------- ---------- ---------- ---------- ----------

615 s ---------- ---------- ---------- ---------- ---------- ---------- ---------- ---------- ----------

64 s ---------- ---------- ---------- ---------- ---------- ---------- ---------- ---------- ----------

658 d ---------- ---------- ---------- ---------- ---------- ---------- ---------- ---------- ----------

658 s ---------- ---------- ---------- ---------- ---------- ---------- ---------- ---------- ----------

662 d ---------- ---------- ---------- ---------- ---------- ---------- ---------- ---------- ----------

662 s ---------- ---------- ---------- ---------- ---------- ---------- ---------- ---------- ----------

757 s ---------- ---------- ---------- ---------- ---------- ---------- ---------- ---------- ----------

768 d ---------- ---------- ---------- ---------- ---------- ---------- ---------- ---------- ----------

768 s ---------- ---------- ---------- ---------- ---------- ---------- ---------- ---------- ----------

796 d ---------- ---------- ---------- ---------- ---------- ---------- ---------- ---------- ----------

796 s ---------- ---------- ---------- ---------- ---------- ---------- ---------- ---------- ----------

798 d ---------- ---------- ---------- ---------- ---------- ---------- ---------- ---------- ----------

798 s ---------- ---------- ---------- ---------- ---------- ---------- ---------- ---------- ----------

8 si ---------- ---------- ---------- ---------- ---------- ---------- ---------- ---------- ----------

805 s ---------- ---------- ---------- ---------- ---------- ---------- ---------- ---------- ----------

811 s ---------- ---------- ---------- ---------- ---------- ---------- ---------- ---------- ----------

92 s ---------- ---------- ---------- ---------- ---------- ---------- ---------- ---------- ----------

94 s ---------- ---------- ---------- ---------- ---------- ---------- ---------- ---------- ----------

230 si ---------- ---------- ---------- ---------- ---------- ---------- ---------- ---------- ----------

454 d ---------- ---------- ---------- ---------- ---------- ---------- ---------- ---------- ----------

332 d ---------- ---------- ---------- ---------- ---------- ---------- ---------- ---------- ----------

454 s ---------- ---------- ---------- ---------- ---------- ---------- ---------- ---------- ----------

621 d ---------- ---------- ---------- ---------- ---------- ---------- ---------- ---------- ----------

64 si ---------- ---------- ---------- ---------- ---------- ---------- ---------- ---------- ----------

621 d male M. patens ---------- ---------- ---------- ---------- ---------- ---------- ---------- ---------- ----------

LC052360.1 A. putorii ---------- ---------- ---------- ---------- ---------- ---------- ---------- ---------- ----------

JX456630.1 E. boehmi ---------- ---------- ---------- ---------- ---------- ---------- ---------- ---------- ----------

KX962352.1 C. plica ---------- ---------- ---------- ---------- ---------- ---------- ---------- ---------- ----------

MF287972.1 C. hepaticum ---------- ---------- ---------- ---------- ---------- ---------- ---------- ---------- ----------

KC753538.1 C. splenaecum ---------- ---------- ---------- ---------- ---------- ---------- ---------- ---------- ----------

KC341985.1 T. vulpis ---------- ---------- ---------- ---------- ---------- ---------- ---------- ---------- ----------

1000 1010 1020 1030 1040 1050 1060 1070 1080

....|....| ....|....| ....|....| ....|....| ....|....| ....|....| ....|....| ....|....| ....|....|

131 s ---------- ---------- ---------- ---------- ---------- ---------- ---------- ---------- ----------

19 s ---------- ---------- ---------- ---------- ---------- ---------- ---------- ---------- ----------

19 si ---------- ---------- ---------- ---------- ---------- ---------- ---------- ---------- ----------

21 s ---------- ---------- ---------- ---------- ---------- ---------- ---------- ---------- ----------

230 s ---------- ---------- ---------- ---------- ---------- ---------- ---------- ---------- ----------

236 s ---------- ---------- ---------- ---------- ---------- ---------- ---------- ---------- ----------

238 s ---------- ---------- ---------- ---------- ---------- ---------- ---------- ---------- ----------

246 s ---------- ---------- ---------- ---------- ---------- ---------- ---------- ---------- ----------

248 d ---------- ---------- ---------- ---------- ---------- ---------- ---------- ---------- ----------

248 s ---------- ---------- ---------- ---------- ---------- ---------- ---------- ---------- ----------

249 s ---------- ---------- ---------- ---------- ---------- ---------- ---------- ---------- ----------

255 s ---------- ---------- ---------- ---------- ---------- ---------- ---------- ---------- ----------

258 s ---------- ---------- ---------- ---------- ---------- ---------- ---------- ---------- ----------

27 s ---------- ---------- ---------- ---------- ---------- ---------- ---------- ---------- ----------

28 s ---------- ---------- ---------- ---------- ---------- ---------- ---------- ---------- ----------

290 d ---------- ---------- ---------- ---------- ---------- ---------- ---------- ---------- ----------

290 s ---------- ---------- ---------- ---------- ---------- ---------- ---------- ---------- ----------

294 s ---------- ---------- ---------- ---------- ---------- ---------- ---------- ---------- ----------

297 s ---------- ---------- ---------- ---------- ---------- ---------- ---------- ---------- ----------

300 s ---------- ---------- ---------- ---------- ---------- ---------- ---------- ---------- ----------

332 s ---------- ---------- ---------- ---------- ---------- ---------- ---------- ---------- ----------

342 s ---------- ---------- ---------- ---------- ---------- ---------- ---------- ---------- ----------

350 d ---------- ---------- ---------- ---------- ---------- ---------- ---------- ---------- ----------

350 s ---------- ---------- ---------- ---------- ---------- ---------- ---------- ---------- ----------

358 s ---------- ---------- ---------- ---------- ---------- ---------- ---------- ---------- ----------

367 s ---------- ---------- ---------- ---------- ---------- ---------- ---------- ---------- ----------

375 d ---------- ---------- ---------- ---------- ---------- ---------- ---------- ---------- ----------

375 s ---------- ---------- ---------- ---------- ---------- ---------- ---------- ---------- ----------

375 si ---------- ---------- ---------- ---------- ---------- ---------- ---------- ---------- ----------

454 s ---------- ---------- ---------- ---------- ---------- ---------- ---------- ---------- ----------

516 s ---------- ---------- ---------- ---------- ---------- ---------- ---------- ---------- ----------

561 s ---------- ---------- ---------- ---------- ---------- ---------- ---------- ---------- ----------

615 d ---------- ---------- ---------- ---------- ---------- ---------- ---------- ---------- ----------

615 s ---------- ---------- ---------- ---------- ---------- ---------- ---------- ---------- ----------

64 s ---------- ---------- ---------- ---------- ---------- ---------- ---------- ---------- ----------

658 d ---------- ---------- ---------- ---------- ---------- ---------- ---------- ---------- ----------

658 s ---------- ---------- ---------- ---------- ---------- ---------- ---------- ---------- ----------

662 d ---------- ---------- ---------- ---------- ---------- ---------- ---------- ---------- ----------

662 s ---------- ---------- ---------- ---------- ---------- ---------- ---------- ---------- ----------

757 s ---------- ---------- ---------- ---------- ---------- ---------- ---------- ---------- ----------

768 d ---------- ---------- ---------- ---------- ---------- ---------- ---------- ---------- ----------

768 s ---------- ---------- ---------- ---------- ---------- ---------- ---------- ---------- ----------

796 d ---------- ---------- ---------- ---------- ---------- ---------- ---------- ---------- ----------

796 s ---------- ---------- ---------- ---------- ---------- ---------- ---------- ---------- ----------

798 d ---------- ---------- ---------- ---------- ---------- ---------- ---------- ---------- ----------

798 s ---------- ---------- ---------- ---------- ---------- ---------- ---------- ---------- ----------

8 si ---------- ---------- ---------- ---------- ---------- ---------- ---------- ---------- ----------

805 s ---------- ---------- ---------- ---------- ---------- ---------- ---------- ---------- ----------

811 s ---------- ---------- ---------- ---------- ---------- ---------- ---------- ---------- ----------

92 s ---------- ---------- ---------- ---------- ---------- ---------- ---------- ---------- ----------

94 s ---------- ---------- ---------- ---------- ---------- ---------- ---------- ---------- ----------

230 si ---------- ---------- ---------- ---------- ---------- ---------- ---------- ---------- ----------

454 d ---------- ---------- ---------- ---------- ---------- ---------- ---------- ---------- ----------

332 d ---------- ---------- ---------- ---------- ---------- ---------- ---------- ---------- ----------

454 s ---------- ---------- ---------- ---------- ---------- ---------- ---------- ---------- ----------

621 d ---------- ---------- ---------- ---------- ---------- ---------- ---------- ---------- ----------

64 si ---------- ---------- ---------- ---------- ---------- ---------- ---------- ---------- ----------

621 d male M. patens ---------- ---------- ---------- ---------- ---------- ---------- ---------- ---------- ----------

LC052360.1 A. putorii ---------- ---------- ---------- ---------- ---------- ---------- ---------- ---------- ----------

JX456630.1 E. boehmi ---------- ---------- ---------- ---------- ---------- ---------- ---------- ---------- ----------

KX962352.1 C. plica ---------- ---------- ---------- ---------- ---------- ---------- ---------- ---------- ----------

MF287972.1 C. hepaticum ---------- ---------- ---------- ---------- ---------- ---------- ---------- ---------- ----------

KC753538.1 C. splenaecum ---------- ---------- ---------- ---------- ---------- ---------- ---------- ---------- ----------

KC341985.1 T. vulpis ---------- ---------- ---------- ---------- ---------- ---------- ---------- ---------- ----------

1090 1100 1110 1120 1130

....|....| ....|....| ....|....| ....|....| ....|....| ...

131 s ---------- ---------- ---------- ---------- ---------- ---

19 s ---------- ---------- ---------- ---------- ---------- ---

19 si ---------- ---------- ---------- ---------- ---------- ---

21 s ---------- ---------- ---------- ---------- ---------- ---

230 s ---------- ---------- ---------- ---------- ---------- ---

236 s ---------- ---------- ---------- ---------- ---------- ---

238 s ---------- ---------- ---------- ---------- ---------- ---

246 s ---------- ---------- ---------- ---------- ---------- ---

248 d ---------- ---------- ---------- ---------- ---------- ---

248 s ---------- ---------- ---------- ---------- ---------- ---

249 s ---------- ---------- ---------- ---------- ---------- ---

255 s ---------- ---------- ---------- ---------- ---------- ---

258 s ---------- ---------- ---------- ---------- ---------- ---

27 s ---------- ---------- ---------- ---------- ---------- ---

28 s ---------- ---------- ---------- ---------- ---------- ---

290 d ---------- ---------- ---------- ---------- ---------- ---

290 s ---------- ---------- ---------- ---------- ---------- ---

294 s ---------- ---------- ---------- ---------- ---------- ---

297 s ---------- ---------- ---------- ---------- ---------- ---

300 s ---------- ---------- ---------- ---------- ---------- ---

332 s ---------- ---------- ---------- ---------- ---------- ---

342 s ---------- ---------- ---------- ---------- ---------- ---

350 d ---------- ---------- ---------- ---------- ---------- ---

350 s ---------- ---------- ---------- ---------- ---------- ---

358 s ---------- ---------- ---------- ---------- ---------- ---

367 s ---------- ---------- ---------- ---------- ---------- ---

375 d ---------- ---------- ---------- ---------- ---------- ---

375 s ---------- ---------- ---------- ---------- ---------- ---

375 si ---------- ---------- ---------- ---------- ---------- ---

454 s ---------- ---------- ---------- ---------- ---------- ---

516 s ---------- ---------- ---------- ---------- ---------- ---

561 s ---------- ---------- ---------- ---------- ---------- ---

615 d ---------- ---------- ---------- ---------- ---------- ---

615 s ---------- ---------- ---------- ---------- ---------- ---

64 s ---------- ---------- ---------- ---------- ---------- ---

658 d ---------- ---------- ---------- ---------- ---------- ---

658 s ---------- ---------- ---------- ---------- ---------- ---

662 d ---------- ---------- ---------- ---------- ---------- ---

662 s ---------- ---------- ---------- ---------- ---------- ---

757 s ---------- ---------- ---------- ---------- ---------- ---

768 d ---------- ---------- ---------- ---------- ---------- ---

768 s ---------- ---------- ---------- ---------- ---------- ---

796 d ---------- ---------- ---------- ---------- ---------- ---

796 s ---------- ---------- ---------- ---------- ---------- ---

798 d ---------- ---------- ---------- ---------- ---------- ---

798 s ---------- ---------- ---------- ---------- ---------- ---

8 si ---------- ---------- ---------- ---------- ---------- ---

805 s ---------- ---------- ---------- ---------- ---------- ---

811 s ---------- ---------- ---------- ---------- ---------- ---

92 s ---------- ---------- ---------- ---------- ---------- ---

94 s ---------- ---------- ---------- ---------- ---------- ---

230 si ---------- ---------- ---------- ---------- ---------- ---

454 d ---------- ---------- ---------- ---------- ---------- ---

332 d ---------- ---------- ---------- ---------- ---------- ---

454 s ---------- ---------- ---------- ---------- ---------- ---

621 d ---------- ---------- ---------- ---------- ---------- ---

64 si ---------- ---------- ---------- ---------- ---------- ---

621 d male M. patens ---------- ---------- ---------- ---------- ---------- ---

LC052360.1 A. putorii ---------- ---------- ---------- ---------- ---------- ---

JX456630.1 E. boehmi ---------- ---------- ---------- ---------- ---------- ---

KX962352.1 C. plica ---------- ---------- ---------- ---------- ---------- ---

MF287972.1 C. hepaticum ---------- ---------- ---------- ---------- ---------- ---

KC753538.1 C. splenaecum ---------- ---------- ---------- ---------- ---------- ---

KC341985.1 T. vulpis ---------- ---------- ---------- ---------- ---------- ---
